# The First Metriorhynchid Crocodylomorph from the Middle Jurassic of Spain, with Implications for Evolution of the Subclade Rhacheosaurini

**DOI:** 10.1371/journal.pone.0054275

**Published:** 2013-01-23

**Authors:** Jara Parrilla-Bel, Mark T. Young, Miguel Moreno-Azanza, José Ignacio Canudo

**Affiliations:** 1 Grupo Aragosaurus-IUCA (Instituto Universitario de Investigación en Ciencias Ambientales de Aragón), Ciencias de la Tierra, Facultad de Ciencias, Universidad de Zaragoza, Zaragoza, Spain; 2 School of Geosciences, University of Edinburgh, Edinburgh, United Kingdom; Ludwig-Maximilians-Universität München, Germany

## Abstract

**Background:**

Marine deposits from the Callovian of Europe have yielded numerous species of metriorhynchid crocodylomorphs. While common in English and French Formations, metriorhynchids are poorly known from the Iberian Peninsula. Twenty years ago an incomplete, but beautifully preserved, skull was discovered from the Middle Callovian of Spain. It is currently the oldest and best preserved metriorhynchid specimen from the Iberian Peninsula. Until now it has never been properly described and its taxonomic affinities remained obscure.

**Methodology/Principal Findings:**

Here we present a comprehensive description for this specimen and in doing so we refer it to a new genus and species: *Maledictosuchus riclaensis*. This species is diagnosed by numerous autapomorphies, including: heterodont dentition; tightly interlocking occlusion; lachrymal anterior process excludes the jugal from the preorbital fenestra; orbits longer than supratemporal fenestrae; palatine has two non-midline and one midline anterior processes. Our phylogenetic analysis finds *Maledictosuchus riclaensis* to be the basal-most known member of Rhacheosaurini (the subclade of increasingly mesopelagic piscivores that includes *Cricosaurus* and *Rhacheosaurus*).

**Conclusions/Significance:**

Our description of *Maledictosuchus riclaensis* shows that the craniodental morphologies that underpinned the success of Rhacheosaurini in the Late Jurassic and Early Cretaceous, as a result of increasing marine specialization to adaptations for feeding on fast small-bodied prey (i.e. divided and retracted external nares; reorientation of the lateral processes of the frontal; elongate, tubular rostrum; procumbent and non-carinated dentition; high overall tooth count; and dorsolaterally inclined paroccipital processes), first appeared during the Middle Jurassic. Rhacheosaurins were curiously rare in the Middle Jurassic, as only one specimen of *Maledictosuchus riclaensis* is known (with no representatives discovered from the well-sampled Oxford Clay Formation of England). As such, the feeding/marine adaptations of Rhacheosaurini did not confer an immediate selective advantage upon the group, and it took until the Late Jurassic for this subclade to dominate in Western Europe.

## Introduction

Crocodylomorpha was a morphologically and ecologically diverse clade during Mesozoic [1]–[5], and one of the first fossil reptile groups to be discovered and studied [6], [7]. During the Mesozoic, the Metriorhynchidae were perhaps the most aberrant members of this clade, being the only archosaurian group to secondarily return to a pelagic lifestyle [8], [9] and evolved numerous adaptations convergent with other marine amniotes (e.g. hydrofoil-like forelimbs, hypocercal tail and large salt glands [10], [11], [12], [13]).

**Figure 1:**
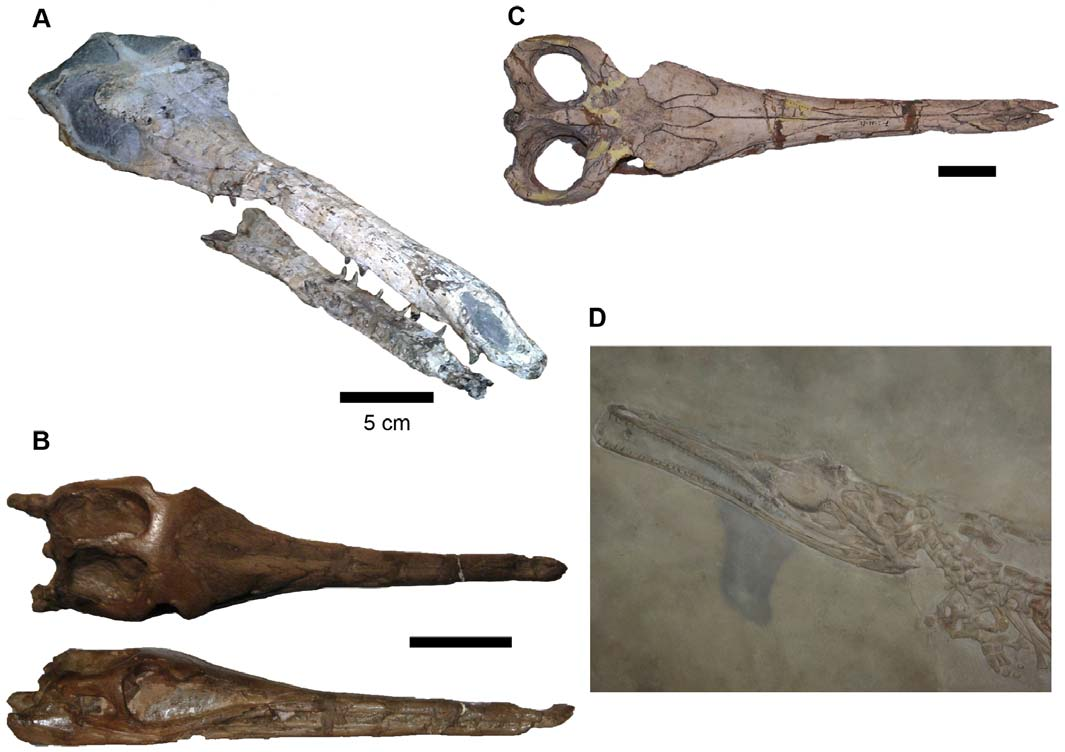
Comparative plate of Rhacheosaurini cranial morphology. A, *Maledictosuchus riclaensis*, holotype MPZ 2001/130a (MPZ, Museo Paleontológico de la Universidad de Zaragoza); B, *Rhacheosaurus gracilis*, referred specimen NHMUK PV R3948 (NHMUK, Natural History Museum, London, United Kingdom); C, *Cricosaurus araucanensis*, holotype MLP 72-IV-7-1; D, *Cricosaurus suevicus*, lectotype SMNS 9808 (SMNS, Staatliches Museum für Naturkunde Stuttgart, Germany). Scale bar = 5 cm.

One of the major metriorhynchid subclades, Metriorhynchinae, consists of numerous small-to-medium bodied species within the genera *Metriorhynchus*, *Gracilineustes*, *Rhacheosaurus* and *Cricosaurus* [14], [15], [16], [17]. There is a temporal and phylogenetic trend towards increasingly specialised piscivory within this group, as more derived and younger taxa had skulls that were less optimized for enduring strong bite forces [14], [16]. The more derived members of Metriorhynchinae are within the subclade Rhacheosaurini (Fig. 1). Rhacheosaurins are not only characterised by having skulls not suited for enduring strong bite forces, but have elongate and tubular snouts, high tooth counts, and uncarinated and unserrated teeth [14], [16], [18] (although three specimens do have carinae: [19], [20]–[21]). Based on this craniodental morphology it has been posited that they were well suited for feeding on small, fast-moving prey [14], [16]. Interestingly, rhacheosaurins also exhibit a temporal and phylogenetic trend towards increasing marine specialisation (e.g. posterior retraction of the external nares, regression of the calcaneum tuber, increase in caudal vertebrae count in the tail fluke [14], [20]). Currently, all discovered species within Rhacheosaurini are known from the Late Jurassic and Early Cretaceous [1], [16], [20], [22], [23], [24].

The origin of Rhacheosaurini is obscure, even though the fossil record of Callovian (final stage of the Middle Jurassic, 164–161 Ma) marine reptiles is noted for its preservation and completeness [25]. During the Callovian, thalattosuchians were highly diverse in Western Europe (in particular England and France) and are also known from South America, India and Dagestan [10], [26], [27], [28], [29], [30], [31], [32]. However, until now no rhacheosaurin specimens have been discovered.

Although the Mesozoic presents a large outcrop in Spain, knowledge of Spanish Mesozoic archosaurs has, unfortunately, been limited. However, in recent years a great effort to document these animals has been made, with the description of an unexpected and diverse fauna of Cretaceous dinosaurs [33]. The Jurassic fossil record is sparse, with the sole exception of the Jurassic-Cretaceous transition which has abundant dinosaur remains [34], [35]. Across the Iberian Peninsula there are exposures of Mesozoic marine sediments; however marine reptiles from these formations are poorly understood [36]. Bardet *et al.* [37] reviewed the Mesozoic marine reptile fossil record of the Iberian Peninsula, and found that plesiosaurs and mosasaurs were the best represented, and that the marine reptile record had a wide temporal range (Middle Triassic–latest Cretaceous). These fossil remains are mainly represented by isolated and fragmentary specimens (vertebrae, jaw fragments and teeth). Teleosaurid and metriorhynchid thalattosuchians are represented in Jurassic deposits from the Iberian Peninsula [38], [39], but few can be identified at the generic level. However, there is a well-preserved skull from the uppermost Toarcian or lowermost Aalenian (Early or Middle Jurassic) of Portugal described as *Pelagosaurus tomarensis* [40] (later re-assigned to *Mystriosaurus* cf. *bollensis* [41]). Ruiz-Omeñaca *et al.* [42] described an isolated ziphodont tooth from Colunga (Asturias, northern Spain) as *Dakosaurus* sp. (recently re-assigned to cf. *Plesiosuchus manselii* [43]). Furthermore, they [43] cite two other thalattosuchian taxa (cf. *Machimosaurus* sp. and Thalattosuchia indet.) in the Upper Jurassic Tereñes Formation.

An incomplete, but exceptionally well preserved skull from Callovian-aged deposits near Ricla (Zaragoza) is also diagnostic. This specimen was discovered by C. Gonzalbo, C. Laplana and M. Soria in 1994 during a prospecting campaign to identify and delimit areas with high numbers of fossils, for either protecting or excavating. This fieldwork was conducted due to the construction of the AVE railway line, which would have made such areas inaccessible. While fragments of bones were preserved in dark limestone nodules, it seems that the skull and three associated vertebrae are an isolated find, as no other vertebrate fossils were discovered at the same level.

This specimen (MPZ 2001/130) is a very famous fossil in the Aragón autonomous community, where it is commonly known as “Cocodrilo de Ricla”, and has been figured numerous times in popular articles and books ([44], [45], [46], [47], [48], [49], [50], [51], [52], [53], [54], [55], [56], [57]). It was deposited in the Museo Paleontológico de la Universidad de Zaragoza (MPZ) soon after its discovery in 1994. Initially the skull was identified as a member of the genus *Metriorhynchus* [44]. More recently, Parrilla-Bel & Canudo [57], in a preliminary study, considered it to be Metriorhynchoidea indeterminate. Until now, this specimen has never been studied in depth.

This specimen has important implications for our understanding of metriorhynchid evolution. First, the exquisite preservation of the skull furthers our knowledge of the geometry and craniofacial form of Callovian metriorhynchids, especially as most Callovian specimens have undergone post-mortem distortion and deformation (see Andrews [10]; Lepage *et al.* [32]). Second, it is the oldest known metriorhynchid from the Iberian Peninsula, and will further elucidate the composition of Jurassic Western European marine ecosystems. Finally, the origins of the metriorhynchid subclade Rhacheosaurini are obscure due to the dearth of specimens from key time stages (such as the Middle Jurassic). Herein we describe a new genus and species, present a revised phylogenetic analysis of the Metriorhynchidae and review the implications this specimen has for the origins of the subclade Rhacheosaurini.

### Geological setting

**Figure 2:**
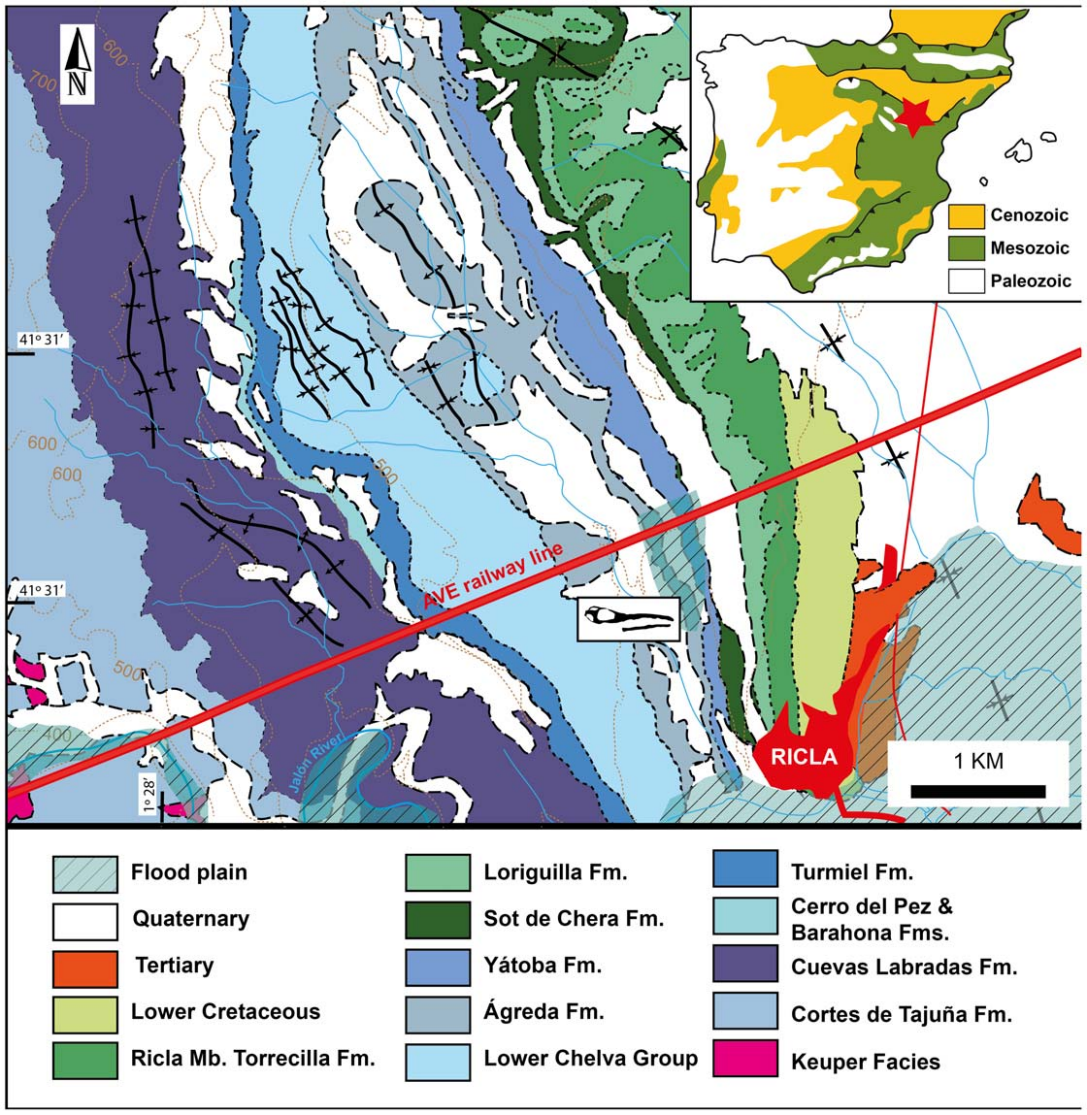
Geographical and geological location of *Maledictosuchus riclaensis* gen. et sp. nov. holotype MPZ 2001/130. Location and geological map of Ricla locality, modified from Lendínez et al. [59]. Abbreviations: Fm., Formation; Fms., Formations; Mb., Member.

The specimen was collected northwest of Ricla (Zaragoza, Spain), in the “*Barranco de la Paridera*” site (“Ricla 2” in [58]). Ricla is located in the north-central area of the Iberian Range (Fig. 2) [59]. The Middle Jurassic strata of this area have been the subject of numerous studies (e.g. [60], [61], [62]). The geology of this area is complex due to the presence of condensed sections [63], [64]. The section where the specimen was collected has been interpreted as belonging to the Domeño Formation (Chelva Group) by some authors [65], whereas others locate it in the Ágreda Formation ([58] and references therein). In any case, the age of the site is safely determined by associated ammonite fossils (see [66] and references therein).

Following the work of Ramajo [58], which is the only work to our knowledge that actually locates the specimen in a stratigraphic column, the specimen is from the Ágreda Formation (contrary to Parrilla-Bel & Canudo [57] who erroneously cited the same source as placing the specimen in the Chelva Formation). Near Ricla this formation consists of alternating muddy and sandy limestones. The Ágreda Formation overlies the Chelva Formation in this region, and lies below the Yátova Formation of Oxfordian age. Its thickness changes through its outcrop, being approximately 90 m deep in the Ricla vicinity. The Ágreda Formation is interpreted as having been deposited in a shallow marine environment that was subtidal to intertidal. The skull was discovered within a level of alternating grey-beige limestones and grey marlstones, in the upper part of the Ágreda Formation. The strata consist of tabular to nodulose, bioclastic and peloidal wackstone to wackstone-packstone facies. The few fossils that are recovered from this level are mainly belemnites, ammonites and bivalves. Bioturbation is abundant, especially at the top of the strata. The specimen was associated with the Middle Callovian ammonite *Erymnoceras coronatum* (thus in the *Erymnoceras coronatum* Sub-Mediterranean ammonite Zone).

## Methods

### Nomenclatural Acts

The electronic edition of this article conforms to the requirements of the amended International Code of Zoological Nomenclature, and hence the new names contained herein are available under that Code from the electronic edition of this article. This published work and the nomenclatural acts it contains have been registered in ZooBank, the online registration system for the ICZN. The ZooBank LSIDs (Life Science Identifiers) can be resolved and the associated information viewed through any standard web browser by appending the LSID to the prefix “http://zoobank.org/”. The LSID for this publication is: urn:lsid:zoobank.org:pub: 0FB0955D-5FAF-47C4-A2BE-114E1DC7D997. The electronic edition of this work was published in a journal with an ISSN, and has been archived and is available from the following digital repositories: PubMed Central, LOCKSS.

### Ethics Statement

All necessary permits were obtained for the described study, which complied with all relevant regulations. The specimen was collected with the authorization of the “Gobierno de Aragón” (Decreto 6/1990, 23 de enero, de la Diputación General de Aragón; Exp: 197/94). We had permission to look at, and photograph, the relevant collection in the Museo Paleontológico de la Universidad de Zaragoza (MPZ). The director of the Museum (José Ignacio Canudo), whose remit includes fossil crocodylians from the MPZ (MPZ 2001/130), is a co-author on this manuscript. None of these specimens were purchased, donated or loaned as part of this study. MPZ 2001/130 forms part of permanent collection of Museum, in which all fossils are publically owned (Gobierno de Aragón).

### Phylogenetic analysis

We undertook a phylogenetic analysis to assess the evolutionary relationships of *Maledictosuchus riclaensis* within Metriorhynchidae. This analysis was based on the taxon and character dataset of Young *et al.* [43]. This resulted in a total of 74 taxa coded for 240 characters, with the non-crocodylomorph pseudosuchian archosaur *Postosuchus kirkpatricki* used as the outgroup taxon. In TNT v1.1 (Willi Hennig Society Edition; [67]) tree-space was searched using new technology search methods in TNT, namely: sectorial search, tree fusion, ratchet and drift; for 1,000 random addition replicates. The default settings for the advanced search methods were changed to increase the iterations of each method per replicate: 100 sectorial search drifting cycles, 100 ratchet iterations, 100 drift cycles and 100 rounds of tree fusion per replicate. This tree-space search procedure was repeated for five different random start seeds. Bremer supports and bootstrap frequencies (1000 bootstrap replicates) were used to assess the robustness of the nodes.

Following Young *et al.* [43] two analyses were carried out. In the first one, all characters were unordered. In the second one, the following characters were ordered: 1, 7, 8, 10, 13, 25, 38, 39, 42, 43, 47, 50, 56, 58, 69, 86, 87, 96, 126, 132, 133, 151, 152, 154, 156, 166, 179, 181, 182, 183, 184, 198, 202, 214, 218, 225, 228, 230, 231 and 237. In both analyses all characters were equally weighted. The data matrix is provided as supplementary material (see Text S1).

## Results

### Systematic Palaeontology

Superorder Crocodylomorpha Hay, 1930 [68] (*sensu* Walker, 1970) [69]

Infraorder Thalattosuchia Fraas, 1901 [70] (*sensu* Young & Andrade, 2009) [1]

Family Metriorhynchidae Fitzinger, 1843 [71] (*sensu* Young & Andrade, 2009) [1]

Subfamily Metriorhynchinae Fitzinger, 1843 [71] (*sensu* Young & Andrade, 2009) [1]

Tribe Rhacheosaurini Young *et al.*, 2011 [16]

#### Type genus

*Rhacheosaurus* von Meyer, 1831 [24]

#### Emended diagnosis

Metriorhynchid crocodylomorphs with the following unique combination of characters (autapomorphic characters are indicated by an asterisk): posterodorsal retraction of external nares, with the anterior margin of the external nares starting posterior to the first premaxillary alveolus (ch. 7∶3)*; development of premaxillary septum that divides the external nares along the skull midline (ch. 9∶1)*; supratemporal fenestra subequal or shorter in length than orbits*; frontopostorbital suture lower than (ventral to) the intertemporal bar (ch. 60∶1)*; infratemporal fenestra shorter in length than the orbit (ch. 86∶2); paroccipital process dorsolaterally orientated, at an approximate 45 degree angle (ch. 104∶1)*; teeth lack carinae (ch. 167∶0); the anterior margin of both angular and surangular bones terminates anterior to the orbit (ch. 139–140∶1)*; presacral vertebrae count increased by one (to 25) (ch. 178∶1)*; number of caudal vertebrae increased by at least eight (>45); humerus shaft greatly reduced, contributing less than 25% of total humeral length (ch. 207∶2); radius and ulna are subequal in size to the radiale and ulnare, respectively; tibia length greatly shortened, measuring less than 30% of femoral length (ch. 225∶3).

#### Phylogenetic definition

The most inclusive clade including *Rhacheosaurus gracilis* von Meyer, 1831 [24], but not *Metriorhynchus geoffroyii* von Meyer, 1832 [72], and *Gracilineustes leedsi* (Andrews, 1913) [10]. Definition from Young *et al.* [16].

*Maledictosuchus* gen. nov.

**ZooBank Life Science Identifier (LSID) for genus.**

urn:lsid:zoobank.org:act:BB05397F-3EA7-42EB-98C9-D6AA3D2EC626

#### Type species

*Maledictosuchus riclaensis* sp. nov.

#### Etymology

“Damned crocodile”. Maledicto, from Latin word *Maledictus*, in reference to all failed attempts to study this fossil.

#### Geological range

Middle Callovian

#### Geographical range

Spain (Europe)

#### Diagnosis

As for the only known species.

*Maledictosuchus riclaensis* sp. nov.

List of synonymies (only the publications figuring the holotype are listed):

1997 *Metriorhynchus* Canudo et al., fig. on p. 20 [44]

1998 “cocodrilo de Ricla” - Canudo, fig. on p. 13 [45]

2000 *Metriorhynchus superciliosus* Blainville, 1853 - Meléndez & Soria-Llop, fig. 7 [46]

2001 *Metriorhynchus* - Meléndez & Molina, fig. 11 [47]

2002 “cocodrilo del Jurásico de Ricla” - Liñán & Rubio, fig. on p. 21 [48]

2003 “metriorrínquido” - Pérez Urresti, fig. on p. 25 [49]

2005 *Metriorhynchus* von Meyer, 1830 - anonymous, fig. on p. 95 [50]

2005 *Metriorhynchus* von Meyer, 1830 - Pérez Urresti, fig. on p. 83 [51]

2006 *Metriorhynchus* von Meyer, 1830 - Gámez Vintaned et al., fig. on p. 12 [52]

2006 *Metriorhynchus* sp. - Liñán Guijarro & Gámez Vintaned, fig. 22 [53]

2010 *Metriorhynchus* - Genera i Monells & Meléndez Hevia, fig. on p. 27 [54]

2010 *Metriorhynchus* von Meyer, 1830 - Liñán, fig. 5 [55]

2010 *Metriorhynchus* von Meyer, 1830 - Sender Palomar et al., fig. 18 [56]

2011 Metriorhynchidae indet. - Parrilla Bel & Canudo, fig. 2 [57]

**ZooBank LSID for species.**

urn:lsid:zoobank.org:act:503A90A7-DEB6-40E6-9CBC-B8A0FC076D5B

#### Holotype

MPZ 2001/130a – an almost complete skull and part of the lower jaw. MPZ 2001/130b, c, d – three vertebrae, preliminary identification is: one cervical, one dorsal, and a third for which the positional identification is indeterminate.

#### Etymology

“Damned crocodile from Ricla”. The specific epithet *riclaensis* denotes the locality where the specimen was found.

#### Type locality and horizon

“Barranco de la Paridera”, Ricla, Zaragoza, Spain. Ágreda Formation. *Erymnoceras coronatum* Sub-Mediterranean ammonite Zone, Middle Callovian, Middle Jurassic [58].

#### Diagnosis

Metriorhynchid crocodylomorph with the following unique combination of characters (autapomorphic characters are indicated by an asterisk): heterodont dentition in which the anterior maxillary teeth are moderately-to-strongly mediolaterally compressed, while the posterior maxillary teeth are subcircular in cross-section; crowns are uncarinated (lack keel). Enamel on labial and lingual surfaces of crowns has ornamentation composed of accessory ridges aligned to the apicobasal axis of the crown. Maxillae hold 30–33 teeth, approximately 18 anterior to the palatines; dentaries have 20–21 teeth adjacent to the mandibular symphysis. Reception pits between the maxillary alveoli, offset slightly laterally on the anterior region of the maxillae and medially on the posterior region of the maxillae, caused by the dentary crowns and reception pits between dentary alveoli caused by the maxillary crowns*. The skull is narrow with a mesorostrine snout (snout contributes 69% of basicranial length). A thin lachrymal anterior process contacts the maxilla, and excludes the jugal from the preorbital fenestra*. Orbits longer than supratemporal fenestra (ch. 42∶2)*. Supratemporal fossae sub-square/sub-circular, with subequal anteroposterior and lateromedial axes (ch. 39∶1). Approximately 60 degree angle formed by the lateral and medial processes of the frontal (ch. 56∶1), with the rostromedial border of the supratemporal fossa (intratemporal flange) rounded. Frontal ornamented with shallow grooves aligned radially (ch. 55∶1). Frontal minimum width between orbits in dorsal view subequal to width of one supratemporal fossa (ch. 57∶1). Palatine has two non-midline anterior processes and a midline anterior process*. Anterior margin of the choanae is ‘W’-shaped with its base directed anteriorly (ch. 101∶3). Basisphenoid with paired ridges located medially on the ventral surface (ch. 113∶1).

### Description and Comparisons

#### Ontogenetic stage and body length estimate

In fossil crocodylomorphs, the progression of vertebral centrum-neural arch fusion is often used as an indicator of ontogenetic stage (e.g. [73]). Neurocentral suture closure in the crocodylian vertebral column follows a consistent pattern, posterior to anterior; with the cervical neurocentral sutures fusing in morphologically mature specimens [73]. The posteroanterior pattern of neurocentral suture closure has been observed in Middle Jurassic metriorhynchids [18]. The vertebrae of *Maledictosuchus riclaensis* (MPZ 2001/130b, c, d) are still under preparation; however, initial inspection suggests that the centra and neural arches are fused.

The basicranial length of MPZ 2001/130a a is approximately 55 cm, which using the body estimation method outlined by Young *et al.* [15] gives a total length of 2.95 m. Compared to other metriorhynchine this specimen had a shorter body length than the species within *Metriorhynchus*, a greater body length than *Rhacheosaurus*, and is comparable in size to the smaller (and basal-most) species in the genera *Cricosaurus* (*C. suevicus* [15] and *C saltillense* [21]) and *Gracilineustes*.

Metriorhynchids exhibit ontogenetic variation in rostrum length, tooth shape, orbit size and temporal fenestra/fossa length [28]. As the orbits are longer than the supratemporal fenestra, it would seem that MPZ 2001/130a is a juvenile. However, basicranial length is similar to adult specimens of other metriorhynchine species (>50 cm); the frontal is fused, with no inter-frontal suture or groove present; the proportion of the preorbital length to basicranial length is high (this ratio becomes more pronounced in adult specimens); and the intertympanic foramen is located in a ventral position, more posterior in juveniles [28]. Based on these lines of evidence, and the fused cervical neural arch-centrum, we consider the holotype of *Maledictosuchus riclaensis* (MPZ 2001/130a) to be a morphologically mature individual.

#### Skull and mandible: general comments

**Figure 3:**
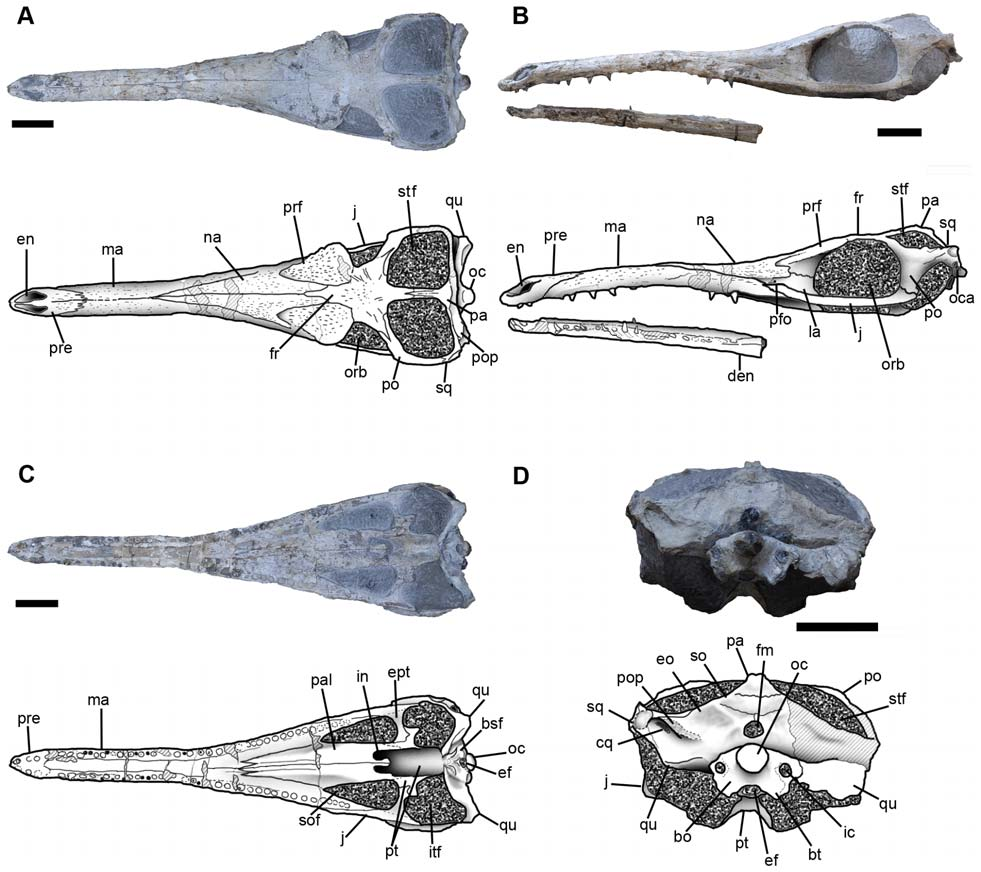
Skull of *Maledictosuchus riclaensis* gen. et sp. nov. holotype MPZ 2001/130a, photographs and interpretative drawings. A, dorsal view; B, left lateral view; C ventral view; D, posterior view. Scale bar = 5 cm. Stripped pattern represents rebuilt mastic surfaces. Dotted pattern represents matrix. Anatomical abbreviations: bo, basioccipital; bsf, basisphenoid; bt, basioccipital basal tubera; cq, cranioquadrate canal; den, dentary; ef, Eustachian foramen; en, external nares; eo, exoccipital; ept, ectopterygoids; fm, foramen magnum; fr, frontal; ic, foramen for the internal carotid artery; in, internal nares; itf, infratemporal fenestra; j, jugal; la, lachrymal; ma, maxilla; na, nasal; oc, occipital condyle; oca, otic canal; orb, orbit; pa, parietal; pal, palatine; pfo, preorbital fossa; po, postorbital; pop, paroccipital process; pre, premaxilla; prf, prefrontal; pt, pterygoids; qu, quadrate; so, supraoccipital; sof, suborbital fenestra; sq, squamosal; stf, supratemporal fenestra.

The holotype (MPZ 2001/130) (Fig. 3) consists of an almost complete skull and part of the lower jaw (MPZ 2001/130a), which were associated with three vertebrae (MPZ 2001/130b, 130c, 130d). While the rostrum is well preserved, the braincase has suffered some erosion, lacking some of the occipital and temporal bones (such as the right supratemporal arch), the posterior processes of the jugals and most of the teeth. Of the lower jaw, only the mandibular symphysis is preserved (dentaries and part of the splenials). In dorsal view, the skull forms an isosceles triangle, with a tapering rostrum without a terminal expansion (such as that observed in teleosaurids, see Andrews [10]). In lateral view the skull is fusiform, with the rostrum lower than the skull table. The rostrum is thin and long. Basicranial length (length from the anterior-most tip of the premaxilla to the posterior-most point of the occipital condyle) is approximately 55 cm, while the rostrum contributes 69% of basicranial length (the length from the tip of the premaxilla to the anterior margin of the orbit is 38 cm). The rostrum is nearly semi-cylindrical, with its lateral walls almost vertical. From the most anterior point of the nasals, the rostrum becomes broader and the skull reaches its maximal width in the posterior part of the supratemporal fossae. In lateral view the rostrum appears slightly curved, ventrally concave, most likely due to deformation.

**Figure 4:**
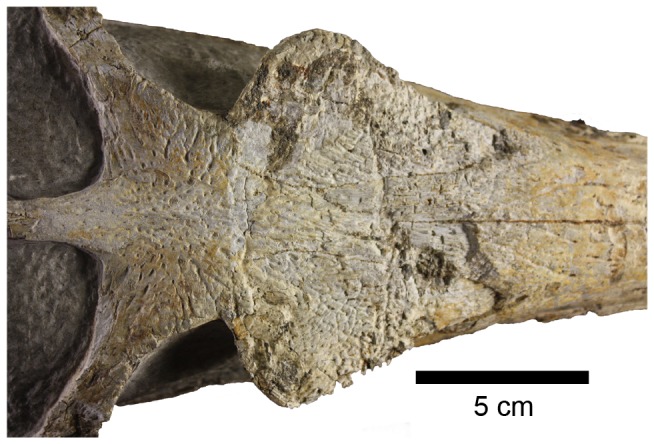
Dorsal view of the frontal region of *Maledictosuchus riclaensis* gen. et sp. nov. holotype MPZ 2001/130a. Notice the ornamentation on the external surfaces of the frontal and the prefrontals. Scale bar = 5 cm.

The dorsal surface of the skull is gently ornamented with a shallow pitted and grooved pattern. The rostrum (premaxilla and maxilla) has a pitted pattern with small elliptical pits; this pattern becomes slightly more packed (i.e. greater density of pits) on the nasals and the prefrontals. The ornamentation on the frontal is slightly deeper, and is composed of radial grooves (Fig. 4). Except for the prefrontals and the frontal, conspicuous ornamentation is absent on the periorbital bones (the lachrymals, jugals and postorbitals).

#### Premaxilla and external nares

**Figure 5:**
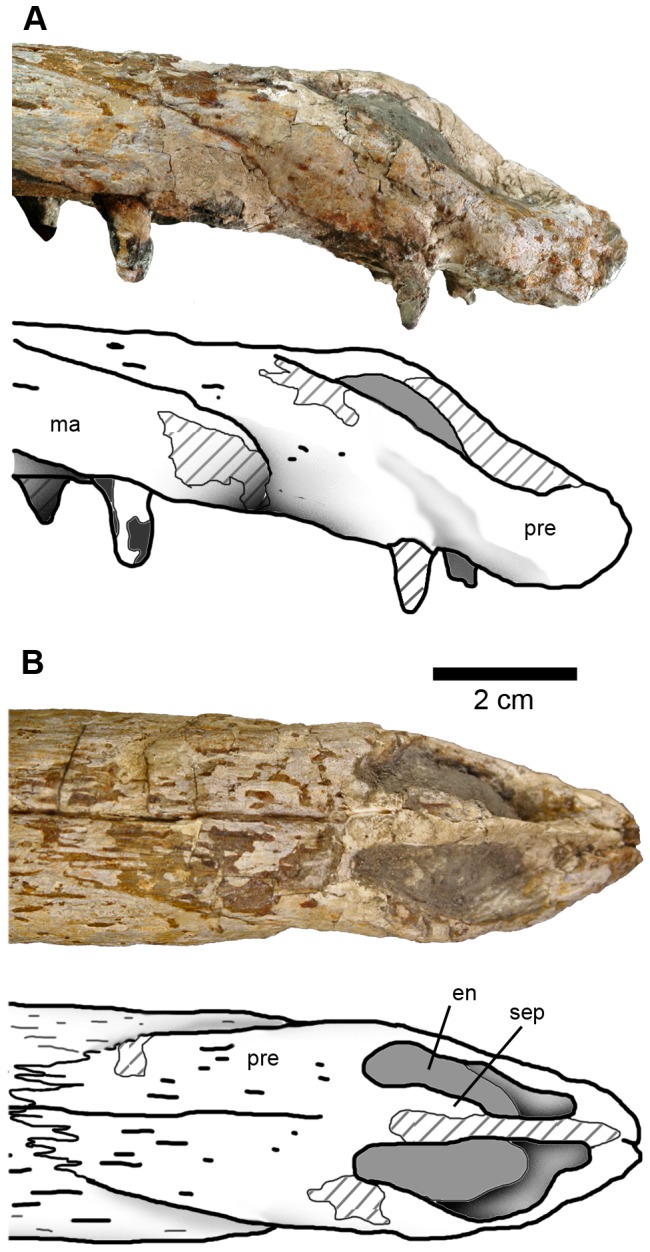
Photographs and interpretative drawings of the premaxilla of *Maledictosuchus riclaensis* gen. et sp. nov. holotype MPZ 2001/130a. A, right lateral view; B, dorsal view. Scale bar = 2 cm. Stripped pattern represents reconstructed surfaces. Anatomical Abbreviations: en, external nares; ma, maxilla; pre, premaxilla; sep, premaxillary septum.

**Figure 6:**
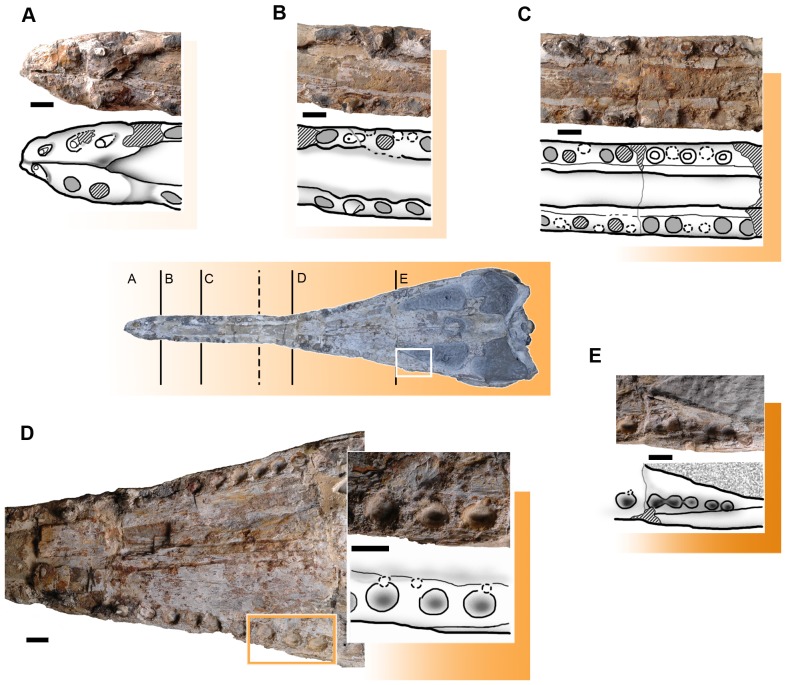
Teeth variation (heterodonty) and reception pits in *Maledictosuchus riclaensis* gen. et sp. nov. holotype MPZ 2001/130a. Photographs and interpretative drawings. A, premaxillary teeth; B, morphology of M1 to M4; C, morphology of M5 to M11; D, morphology of M15 to M26, and detailed photograph and drawing of M23 to M25; E, morphology of M26 to M31. Stripped pattern represents rebuilt mastic surfaces and teeth; grey colored circles represent empty alveoli (teeth are not preserved); circles with broken line represent reception pits. Scale bar = 1 cm.

The premaxillae are 9 cm long along the midline, contributing to 16% of basicranial length, and completely enclose the external nares (Fig. 5). Ornamentation on the external surface is composed of numerous shallow, small elliptical pits and short, fine grooves. Both premaxillae bear three alveoli (Fig. 6), as with all other metriorhynchids (e.g. [1], [10], [11], [14]). The alveoli are separated by intervals of approximately 5 mm. There are no complete premaxillary teeth. On the left ramus, the teeth are partially preserved. All preserved teeth are procumbent (i.e. the crowns have a slightly forward orientation). Between the premaxillary and maxillary tooth rows there is a diastema of approximately 2 cm.

The maxillary process ( = posterodorsal process) of the premaxilla projects posteriorly, terminating level to the third maxillary alveolus. The premaxilla-maxilla suture is strongly interdigitated dorsally and runs anteroventrally on the lateral surface. There is a slight constriction at the contact between both bones in the ventrolateral region. In palatal view the preservation of the premaxilla is poorer.

Most metriorhynchids have a single anterodorsally orientated external naris, such as *Metriorhynchus superciliosus* (e.g. NHMUK PV R6859. NHMUK, Natural History Museum, London, United Kingdom), *Gracilineustes leedsi* (e.g. NHMUK PV R3014) and “*Metriorhynchus*” *brachyrhynchus* (NHMUK PV R3804). The members of the subclade Rhacheosaurini have a different morphology: the external naris is divided by a premaxillary midline septum, and is anterodorsally and laterally oriented (e.g. *Rhacheosaurus gracilis* NHMUK PV R3948; *Cricosaurus suevicus* SMNS 9808; SMNS, Staatliches Museum für Naturkunde Stuttgart). We consider it likely that *Maledictosuchus riclaensis* shares the divided, anterodorsally and laterally orientated narial morphology; however, due to preservation, the internarial bar ( = premaxillary septum; [14]) is incomplete. The internarial bar was reconstructed after preparation; however, the posterior part of the bar is real (Fig. 5). The nares are approximately 3 cm in length. In relation to the tooth-row, they begin after the first premaxillary alveolus, while its posterior margin continues posteriorly past the final premaxillary alveolus terminating before the first maxillary alveolus.

#### Maxilla

Maxillae are very large bones which form the greater part of the rostrum. Ornamentation on the external surface is composed of numerous small elliptical pits and discontinuous grooves oriented anteroposteriorly. The maxillae suture along the dorsal midline, separating the nasals from the premaxillae. This suture is 7 cm long (12% of basicranial skull), anteriorly delimited by the maxillary processes of the premaxillae and posteriorly delimited by the anterior angle of the nasals (Fig. 3A).

**Figure 7:**
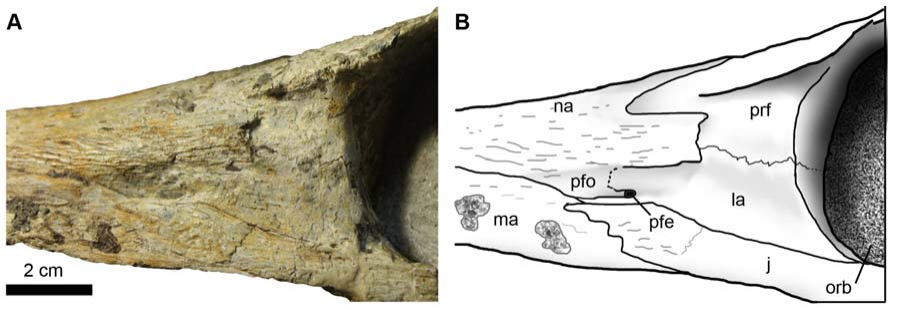
Left preorbital region of *Maledictosuchus riclaensis* gen. et sp. nov. holotype MPZ 2001/130a in lateral view. Scale bar = 2 cm. Anatomical abbreviations: j, jugal; la, lachrymal; ma, maxilla; na, nasal; orb, orbit; pfe, preorbital fenestra; pfo, preorbital fossa; prf, prefrontal.

In lateral view, the maxillae terminate prior to the anterior margin of the orbits, and their greatest length is along the alveolar border. The alveolar border is arched ventrally, but this is probably due to deformation of the rostrum, and it extends in ventral view from the premaxillae to the jugals (Fig. 3C). The maxillae contact the nasals along their posterodorsal border; the sutures are marked by a shallow groove. Ventral to the nasals, the maxillae contact the lachrymals and the jugals. The maxillae form the anteroventral part of the preorbital fossae (Fig. 7).

**Figure 8:**
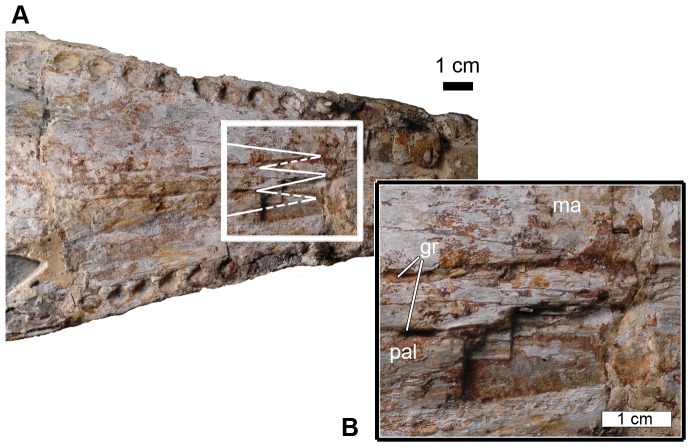
Palate of *Maledictosuchus riclaensis* gen. et sp. nov. holotype MPZ 2001/130a. A, general view of the posterior region, with the suture drawn in white; B, detail of the maxilla-palatine suture. Scale bar = 1 cm. Anatomical Abbreviations: gr, palatal-maxillary groove; ma, maxilla; pal, palatine.

In palatal view, the maxillae are poorly preserved. However, the maxillae suture along the midline forming part of the secondary palate. Posteriorly, the maxillae contact the palatines. The maxilla-palatine suture is ‘W’-shaped, and is orientated posteriorly (Fig. 8). This suture continues posterolaterally, with the maxillae forming the anterolateral border of the suborbital fenestrae (Fig. 3C).

**Figure 9:**
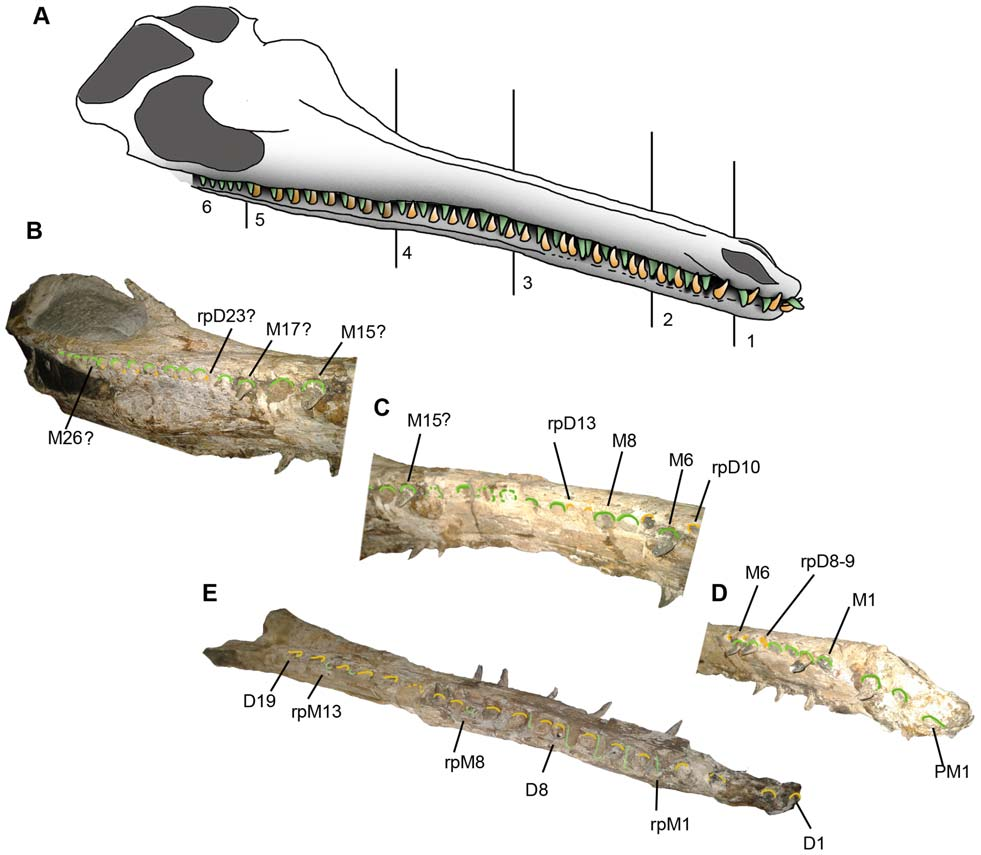
Reconstruction of the occlusion mechanism of *Maledictosuchus riclaensis* gen. et sp. nov. holotype MPZ 2001/130a. A, interpretative drawing of the occlusion mechanism. The interpretation has been created from morphology, size and position of teeth/alveoli and reception pits. The divisions 1 to 6 correspond to: (1) premaxilla, and (2–6) subdivisions of the maxilla. B, Right anteroventral view of zones 5–6. C Right anteroventral view of zones 3–4. D, Right anteroventral view of zones 1–2. E Right anterodorsal view of dentary. Abbreviations: D, dentary tooth; M, maxillary tooth; PM, premaxillary tooth; rpD, reception pit produced by dentary tooth; rpM, reception pit produced by maxillary tooth. Green colour: teeth/alveoli of maxilla and their reception pits on the dentary; Yellow: dentary teeth/alveoli and their reception pits in maxilla.

In a previous paper, 34 alveoli were counted [57], but each maxilla has between 30 and 33 alveoli (the posterior region of the maxillae are poorly preserved and the alveoli are hard to identify). Unfortunately, the specimen only has two completely preserved teeth. The morphology of the alveoli varies along the tooth row (Fig. 6). Four regions are distinguished: the four anterior-most alveoli (M1–M4) are larger and mediolaterally compressed, with a labiolingual width of approximately 5 mm, and a mesiodistal length of 8 mm. The long axis of these alveoli forms an angle of approximately 155 degrees with the long axis of the skull (Figs. 6B, 9A: zone 2). At the mid-snout, from M5 to approximately M11, the alveoli are subcircular and alternate with small ‘depressions’ that are offset laterally. These reception pits (formed by the corresponding dentary crown) are not aligned with the alveolar row (Figs. 6C, 9A: zone 3). Posteriorly, from M12 to M16, there is a region badly preserved (Fig. 9A: zone 4). From M17 to M25, the alveoli are subcircular and subequal in size with an average alveolar diameter of 7 mm and a constant interalveolar space of 3 mm. In that region, small reception pits are found medially to the alveoli (Figs. 6D, 9A: zone 5). The five posterior-most alveoli (M26–M30) are smaller, circular, and very closely packed, with an alveolar diameter of approximately 4–5 mm (Figs. 6E, 9A: zone 6). As the ventral surface of the rostrum is concave, the teeth are not horizontally aligned, with the posterior-most maxillary alveoli on the same plane as the premaxilary alveoli. However, as stated above, the snout has experienced post-mortem deformation, so this is unlikely to be an in vivo morphology.

**Figure 10:**
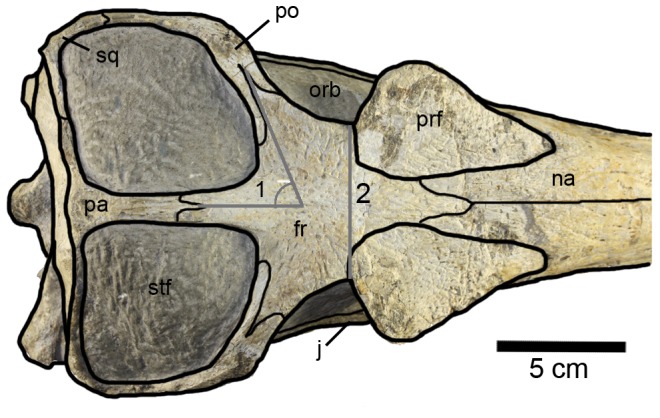
Posterior region of *Maledictosuchus riclaensis* gen. et sp. nov. holotype MPZ 2001/130a in dorsal view. Scale bar = 5 cm. Anatomical abbreviations: fr, frontal; j, jugal; na, nasal; orb, orbit; pa, parietal; po, postorbital; prf, prefrontal; sq, squamosal; stf, supratemporal fenestra; 1, angle between medial and lateral frontal processes; 2, frontal minimum width between orbits.

Nasals. The nasals are paired, unfused elements. The external surface of the nasals (like maxillae) is ornamented with shallow grooves and pits. In dorsal view the nasals are subtriangular (like all thalattosuchians; e.g. [10]), broad, and extensively contribute to the upper surface of the rostrum (Fig. 3A, B). Along their anterior margin the nasals contact the maxillae; thus being separated from the premaxillae. Along their posterior margin the nasals contact the frontal, this suture tapers to a point, forming a ‘V’-shape pointing anteriorly. This suture forms an angle of approximately 12 degrees with anteroposterior axis of the skull and bends posteriorly to the prefrontals ( = dorsoposterior processes) (Fig. 10). The interdigitated nasal-prefrontal suture extends anterolaterally on the dorsal surface of the snout and curves posteriorly on the lateral surface.

The nasals are broadly exposed on the lateral surface of the skull, forming an acute process beneath the prefrontals ( = lateroposterior processes) (Fig. 7). The lateroposterior processes terminate posterior to the preorbital fossae and form the dorsal margin of the preorbital fossae. These processes contact the lachrymals ventrally and the prefrontals dorsally.

#### Lachrymals

The lachrymals are only exposed on the lateral surfaces of the rostrum, in front of the orbits. This is a consequence of the lateral expansion of the nasals and prefrontals, a morphology that is characteristic of all metriorhynchids [10], [11], [14], [74]. The lachrymals are concave with a lightly ornamented external surface with very shallow grooves (i.e. no conspicuous ornamentation). The posterior margins of the lachrymals contribute to the anterior margins of the orbits, forming the lower third. There are sutural contacts with the both the prefrontals and nasals dorsally, with the jugals ventrally, and a small contact with the maxillae anteriorly (Fig. 7).

Along the lachrymal-prefrontal contact there is an anteroposteriorly orientated crest or ridge (best preserved on the right side). This ridge lies within a depressed area, anterior to the orbit but posterior to the preorbital fenestra. Young & Andrade [1] named this the lachrymal-prefrontal fossa. These fossae are restricted to the concave, lateral surfaces of the lachrymals and the prefrontals. Both the fossae and the crests are metriorhynchid apomorphies [1].

Anteriorly, the lachrymals contact the maxillae. The contact between these elements is very small, and is approximately level to the anterior termini of the jugal anterior processes. In other metriorhynchids (e.g. *Geosaurus giganteus*, *Cricosaurus araucanensis*; see figures in Young & Andrade [1]) the lachrymals are excluded from the maxillae by the preorbital fossae and the nasal lateroposterior processes (i.e. not by a jugal–nasal contact). However, in *Maledictosuchus riclaensis* there is a thin anterior process of the lachrymal that continues anteriorly, ventral to the preorbital fenestra. This anterior process contacts the jugal along its ventral margin. This process results in a contact between the lachrymal and the maxilla, which excludes the jugal from the preorbital fenestra, and limits the involvement of the jugal with the preorbital fossa (Fig. 7). A similar arrangement occurs in *Cricosaurus schroederi* ([1]: figure 5b). However, instead of thin lachrymal anterior processes, in this species these processes are wide, much wider than the jugal anterior processes. These broad lachrymal anterior processes results in much more extensive contact between the lachrymals and maxillae in *C. schroederi* [75].

#### Prefrontals

As with all other metriorhynchids, the prefrontals are enlarged laterally and overhang the orbits [10], [11], [14]. These elements have two distinct regions: an upper portion, which extends over the lateral margin of the orbit, and a downwardly deflected region. This upper region overhangs the anterior third of the orbit (overhanging by approximately 50% of the width of the prefrontal). This overhang is extensive, as in dorsal view, it exceeds the jugal bar laterally (Figs. 3, 10). The dorsal ( = external) surface of this upper region is ornamented with pits and short shallow grooves (Fig. 4).

In dorsal view, the prefrontals are teardrop shaped with the apex directed anteriorly, and the inflection point on the outer (lateral) margins project posteriorly, forming an angle of approximately 75 degrees to the anteroposterior axis of the skull. In dorsal view the prefrontals are twice as long as wide; their maximal length is 10 cm, while their maximal width is 5 cm. The medial borders of the upper region contact the frontal posteriorly and the nasal anteriorly (Fig. 10). The anterior end of the prefrontals fits between the dorsoposterior and lateroposterior nasal processes (see Fig. 3C). The nasal-prefrontal suture extends anterolaterally on the dorsal surface and curves posteriorly on the lateral surfaces of the snout. The nasals terminate level to the anterior border of the lachrymal-prefrontal fossae. The prefrontal-frontal sutures start at approximately the anterior third of the orbit dorsal margins. These sutures run straight from their respective orbital margin, and are almost perpendicular to the anteroposterior axis for about 1.6 mm, and then they curve anteriorly.

#### Frontal

The external surface is slightly concave and has a more conspicuous ornamentation pattern than the other cranial bones, being composed of subcircular pits and radial grooves (Fig. 4). Anteriorly, the frontal terminates in a wedge-shaped process ( = anteromedial process) that extends between the nasal dorsoposterior processes. This suture forms a 12 degree angle with the sagittal plane and bends posteriorly to contact the prefrontals. Posterior to the naso-frontal suture, the frontal contacts the prefrontals. Posteriorly the frontal has a medial process that contacts the parietal, and two lateral processes that contact the postorbitals. The participation of the frontal in the dorsal margin of the orbits is reduced due to the expansion of the prefrontals and the postorbitals. The minimum interorbital distance across the frontal is 6 cm, while the maximal is 10 cm (Fig. 10).

The suture between the frontal lateral processes and the postorbitals is a posterolaterally directed V-shape. These lateral processes constitute the posterior margins of the orbits and the anterior margins of the supratemporal fenestrae. The medial process of the frontal contacts the parietal forming the intertemporal bar ( = frontoparietal bar) between supratemporal fossae. The frontoparietal suture is interdigitating with a central V–shape pointing anteriorly. This suture is located slightly anterior to the midpoint of the intertemporal bar. The lateral and medial processes of the frontal form an angle of 60 degrees with one another (Fig. 10).

#### Squamosals

Only the left squamosal is preserved; however, its dorsal surface is partially eroded. The squamosal forms the posterolateral border of the supratemporal fossa (Figs. 3, 10). The anterior process of the squamosal is short, and contacts the postorbital forming the posterior part of the supratemporal arch. This arch is markedly narrow, a characteristic of all thalattosuchians (e.g. [10]). Along its posteromedial edge ( = medial process), the squamosal contacts the parietal. The medial process is orientated slightly posterolaterally, and is briefly exposed on the occipital surface of the skull. The medial and lateral processes of the squamosal meet at a nearly right angle (forming the posterolateral corner of the supratemporal fossa).

**Figure 11:**
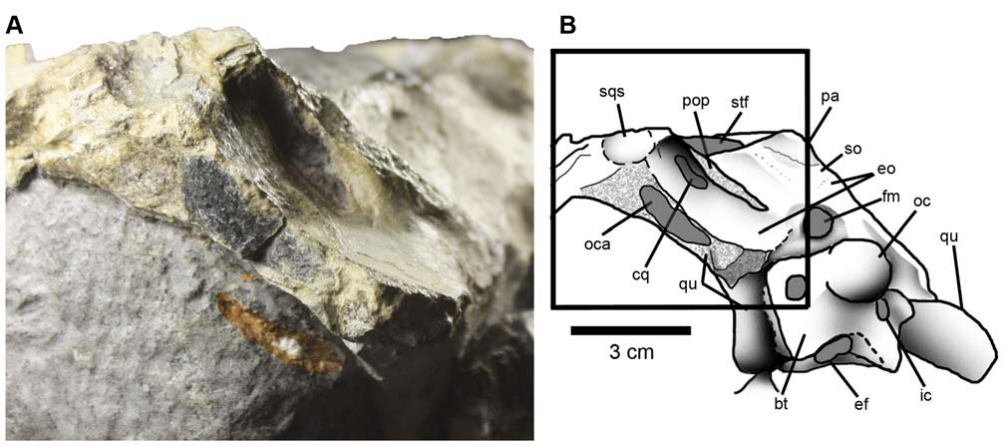
Left posterolateral region of *Maledictosuchus riclaensis* gen. et sp. nov. holotype MPZ 2001/130a. A, detailed photograph of the posterolateral region; B, interpretative drawing of the posterolateral region. Scale bar = 3 cm. Dotted pattern represents broken regions. Anatomical abbreviations: bt, basioccipital basal tubera; cq, cranioquadrate canal; ef, Eustachian foramen; eo, exoccipital; fm, foramen magnum; ic, foramen for the internal carotid artery; oc, occipital condyle; oca, otic canal; pa, parietal; pop, paroccipital process; qu, quadrate; so, supraoccipital; sqs, squamosal flat surface; stf, supratemporal fenestra.

The boundaries between the squamosal and the parietal, exoccipital and quadrate are unclear; the sutural contacts are missing either due to fusion or poor preservation. The squamosal has a distinct lateral subcircular surface, which is slightly anterolateral to the paroccipital process (Fig. 11). This surface is smooth and slightly concave, and is orientated posterolaterally (Fig. 11). This morphology is also present in *Cricosaurus araucanensis*, *Metriorhynchus* and *Dakosaurus andiniensis* [74].

#### Parietal

**Figure 12:**
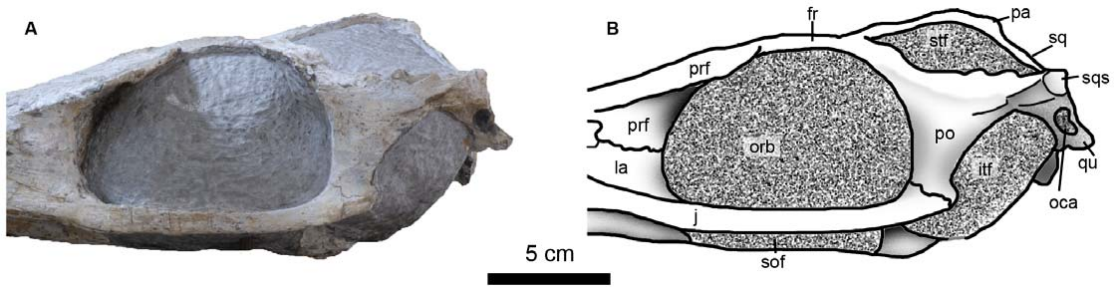
Posterior region of *Maledictosuchus riclaensis* gen. et sp. nov. holotype MPZ 2001/130a in lateral view. Scale bar = 5 cm. Anatomical abbreviations: fr, frontal; itf, infratemporal fenestra; j, jugal; la, lachrymal; oca, otic canal; orb, orbit; pa, parietal; po, postorbital; prf, prefrontal; qu, quadrate; sof, suborbital fenestra; sq, squamosal; sqs, squamosal flat surface; stf, supratemporal fenestra.

The parietal, as with all crocodylomorphs, has fused into a single element [74] and forms the posterior and medial margins of the supratemporal fossae, and together with the frontal constitutes the intertemporal bar. The parietal constitutes slightly more than 50% of the intertemporal bar. The intertemporal bar becomes narrower along its length, so that the frontal contribution is noticeably wider than the parietal contribution (Fig. 10). In lateral view the intertemporal bar is convex, higher than the anterior region of the frontal, and is the dorsal-most region of the skull (Fig. 12). The anterior process of the parietal is narrow and has a midline longitudinal groove on the dorsal surface, which could be evidence of a midline suture (CT scanning will be needed to confirm this). Posteriorly, the anterior process becomes noticeably wider, and in dorsal view is triangular in shape with a convex posterior margin (Fig. 10). The parietal has two lateral processes that contact the squamosals, however, the sutures are difficult to observe. In occipital view, the parietal contacts the supraoccipital along its ventral margin (Fig. 3D).

#### Supratemporal fossae/fenestrae

Due to the infill of matrix, the supratemporal fenestrae are obscured. The supratemporal fossae are large, forming most of the skull roof, and are subtrapezoidal in shape. The margins of each fossa are formed anteriorly by the frontal, anterolaterally by the postorbital, posterolaterally by the squamosal and posteromedially by the parietal (Fig. 10). The intratemporal flange [76] cannot be observed due to matrix. The fossae are slightly shorter than the orbits. The longest axis is anteromedially - posterolaterally directed and is 10 cm long; the shortest axis is perpendicular to the longest axis and is 7 cm long. The medial margins are 7.5 cm long, and the posterior margins are 7 cm long. The minimum distance between the fossae is located in the posterior third of the intertemporal bar, and is less than 1 cm. Both the posterolateral and posteromedial corners are rounded, and form acute and obtuse angles, respectively; the anteromedial and anterolateral corners form angles of approximately 60 and 120 degrees, respectively. The squamosal-parietal bar (forming the posterior margin) is slightly oriented posterolaterally, and it is lower than the intertemporal bar (Fig. 12).

#### Preorbital fossae/fenestrae

Herein we follow hypothesis two of Fernández & Herrera [77], in which the antorbital cavities are internalised in metriorhynchids. The openings classically referred as the “antorbital fenestrae” in this clade is in fact neomorphic preorbital openings for the excretion of salt. These openings are connected via ducts to a chamber that housed large salt-glands (see [12], [13], [77], [78]). Both preorbital fenestrae are bound by an elongate, narrow and obliquely orientated fossa, which in turn are bound by the jugal, the lachrymal, the nasal and the maxilla. The fenestrae themselves are bound by the lachrymals and the nasals (Fig. 7).

#### Orbits

The orbits are oval, large, and are orientated laterally. This is in contrast to the smaller, dorsolaterally oriented orbits of teleosaurids (e.g. *Steneosaurus leedsi*; [10]), and the smaller slightly more lateral orbits of basal metriorhynchoids (e.g. *Teleidosaurus calvadosii*; NHMUK PV R2681). With a height of 6.5 cm, and maximal length of 10 cm, the orbits are larger than the supratemporal fossae (7.5 cm long). The lengths of the orbits are approximately 18% of basicranial length. As with all other metriorhynchids, the prefrontals expand laterally over the dorsal rim of the orbits (e.g. [10], [11], [14]). The anterior rim of the orbits are bound by the prefrontal and the lachrymal; the ventral rim is exclusively bound by the jugal; the posterior rim is bound by the postorbital, and slightly by the ascending process of the jugal; and the dorsal rim is bound by the prefrontal, frontal and the postorbital (Fig. 12). As there is still matrix within the orbits, we cannot determine the presence of sclerotic rings. In the Callovian basal metriorhynchine *Metriorhynchus superciliosus* the sclerotic rings are rarely preserved and never complete (e.g. GLAHM V983, GLAHM V987; Hunterian Museum, Glasgow, United Kingdom).

#### Infratemporal fenestrae

The infratemporal fenestrae ( = laterotemporal fenestrae) are not well preserved. The quadratojugals and the posterior process of the jugals, which together bound the fenestrae ventrolaterally, are missing. Although these bones are missing, the extent of the fenestrae seems to be small in comparison with the orbits: their length would have been less than half of the orbital length. In addition, their height would also have been shorter than the orbital height (Fig. 12).

#### Jugals

The jugals are incomplete in MPZ 2001/130a. The posterior processes of both left and right jugals are missing. The ascending processes are very short, being largely indistinguishable from the main body of the jugal. The ascending processes contact the postorbitals (Fig. 12). The anterior processes are slender and slightly compressed. Under the orbits, the jugals are distinctly laterally compressed with an almost rectangular cross section. Under the right orbit, the jugal is slightly undulate, probably due to deformation. Anterior to the orbits, the anterior processes are very slightly sigmoidal, although not as strongly sigmoidal as *Dakosaurus andiniensis* [74]. The anterior processes extend further anteriorly than the prefrontals and the lachrymals (Fig. 7). The lachrymals contact the dorsal margin of the jugal anterior processes, while the ventral margin contacts the maxillae. The jugals lack conspicuous ornamentation and neurovascular foramina.

#### Quadratojugals

Both of the quadratojugals are missing.

#### Postorbitals

Both postorbitals are preserved. In lateral view (Fig. 12), both postorbitals are ‘T’-shaped, being composed of the frontal process, squamosal process and the postorbital bar ( = descending process). The frontal process is anteromedially directed, contacts the lateral process of the frontal and forms the anterolateral part of the supratemporal fossa. The squamosal process of the postorbital forms the lateral part of the supratemporal arch, and contacts the squamosal. When observed in lateral view, the postorbital is strongly arched, making it noticeably concave on its dorsal surface. This results in the supratemporal arch being significantly lower than the intertemporal bar.

The postorbital descends ventrally to form the postorbital bar, and contacts the jugal through an undulating suture that is posteroventrally to anterodorsally directed. The external surface of the postorbital bar lacks ornamentation. The upper part of the postorbital bar projects laterally, and together with the frontal processes constitutes the widest part of the cranium. On the lateral surface of the postorbital bar there is a ridge that extends onto the squamosal process.

#### Supraoccipital

The supraoccipital is only exposed on the occipital surface of the skull. Its external or occipital surface is slightly concave. The supraoccipital contacts the parietal dorsally (with a horizontal dentate suture) and the exoccipital ventrally by a dentate suture. It is excluded from participating in the foramen magnum by the exoccipital (Fig. 3D). Post-temporal openings ( = post-temporal fenestra; post-temporal foramen) on the occipital surface of the skull are not present. The size and presence of these openings are variable in metriorhynchids, even within species (see [79]: 373).

#### Exoccipital

The occipital surface of the skull is broken, with a large arched crack occurring dorsal to the occipital condyle. The right portion of the exoccipital has not been preserved and has been reconstructed. Whether due to preservation or fusion, the sutures between the exoccipital and the basicranium are difficult to observe. Dorsally, the exoccipital contacts the supraoccipital by a gently concave dentate suture, and laterodorsally it contacts the squamosals and parietal (Fig. 3D). There is a gentle rim below the exoccipital-supraoccipital suture, just above the foramen magnum. Ventrally, the exoccipital would have contacted the basioccipital. However, due to post-mortem damage the sutures are unclear, and it seems that the exoccipital was excluded from the occipital condyle.

The exoccipital forms the greatest part of the occipital surface of the skull. Dorsal to the paroccipital processes, it is slightly concave. The left (and only preserved) paroccipital process is broken, but it would have been orientated dorsolaterally at an approximate 45 degree angle (Fig. 3D). This is very different from that observed in other Callovian metriorhynchids, such as *Metriorhynchus superciliosus* (e.g. NHMUK PV R6859) and *Gracilineustes leedsi* (e.g. NHMUK PV R3014) as they have horizontal paroccipital processes. The skulls referred to “*Metriorhynchus*” *brachyrhynchus* are generally very strongly dorsoventrally compressed, but the left paroccipital process of NHMUK PV R3804 has a slight dorsolateral orientation. Dorsolaterally orientated paroccipital processes are a characteristic of the subclade Rhacheosaurini (see supplementary material and appendices of Young & Andrade [1]; Young *et al.* [16]). The ventral surface of the paroccipital process forms a crest that overhangs a recess for the cranioquadrate canal. The cranioquadrate canal is elongated and obliquely oriented (Fig. 11). The exoccipital extends ventrolaterally to this canal and contacts the quadrate. The cranioquadrate canal is separated from the otic canal by the exoccipital, quadrate and squamosal, a condition also observed in other metriorhynchids (*Dakosaurus*, *Cricosaurus* and *Metriorhynchus*; [74], [79], [80]). Additionally, the exoccipital bears the foramen magnum, which is partially preserved. Its maximum width is 13 mm. Ventrolateral to the occipital condyle there are two large foramina (for the internal carotid arteries) with a diameter of approximately 7 mm (Fig. 3D). Preservation makes determining the positions of the foramina for the cranial nerves difficult.

#### Quadrate

**Figure 13:**
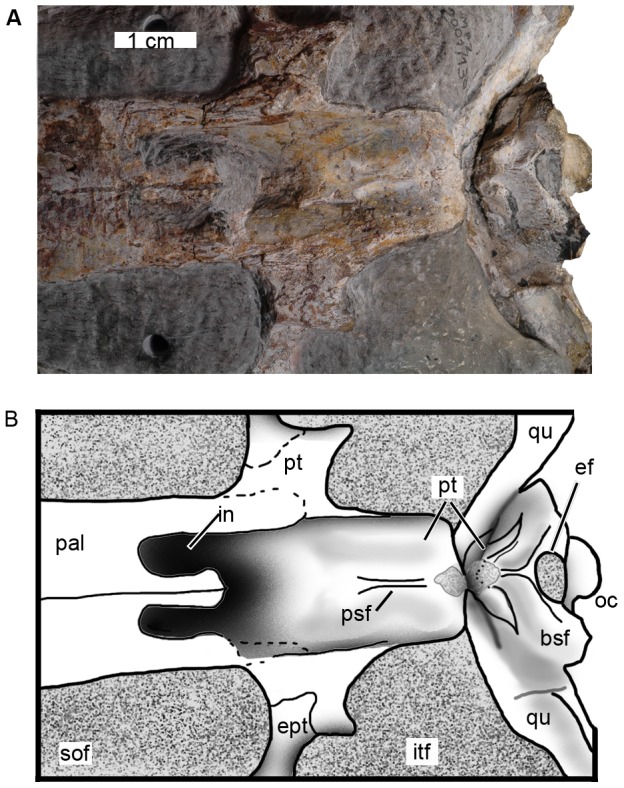
Posterior region of the palate of *Maledictosuchus riclaensis* gen. et sp. nov. holotype MPZ 2001/130a. Scale bar = 1 cm. Anatomical abbreviations: bsf, basisphenoids; ef, Eustachian foramen; ept, ectopterygoids; in, internal nares; itf, infratemporal fenestra; oc, occipital condyle; pal, palatine; psf, parasphenoids; pt, pterygoids; qu, quadrate; sof, suborbital fenestra.

The left quadrate is nearly entirely missing and the right quadrate is not complete (Fig. 3D). The quadrate contacts the squamosal dorsally, the ventrolateral region of the exoccipital posterodorsally, and closes the otic aperture (external auditory opening). This region is best preserved on the left side (Fig. 11). In ventral view, the quadrate contacts the pterygoid and basisphenoid (Fig. 13).

#### Basioccipital

The basioccipital forms the ventromedial part of the occiput. Due to the fracturing in this region, the sutures are unclear. However, it would have contacted the exoccipital dorsolaterally. As the sutures of basioccipital-exoccipital contact are unclear, it seems as though the occipital condyle is formed solely by the basioccipital. The occipital condyle is subcircular in occipital view, and its neck is distinct, short and perpendicular to the occipital plane. The basioccipital has two processes that extend ventrolaterally that form the basal tubera (Fig. 3D). Between them, the ventral margin is deeply notched along the midline. Anterior to this is the Eustachian ( = pharyngotympanic) foramen (Fig. 11).

#### Basisphenoid

The basisphenoid contacts the basioccipital posteriorly, and forms the anterior part of the Eustachian foramen (Fig. 13). The walls of the foramen converge anteriorly on the mid-line forming a small double ridge. Anteriorly and laterally, the basisphenoid unites with the pterygoids.

#### Palatines and internal nares

The palatines are exposed on the palatal surface of the skull. They suture along the skull midline and together with the palatal shelves of the premaxillae and maxillae form the secondary palate. The palatines have two lateral non-midline anterior processes in addition to a midline anterior process. The two non-midline anterior processes are a metriorhynchine apomorphy [1]; however, *Maledictosuchus riclaensis* is the only known specimen that has these two lateral processes and a third midline process as well (Fig. 8). The lateral, non-midline, processes originate at the anterior border of the suborbital fenestrae, and remain separate from the alveolar borders. Anteriorly, the three processes meet the palatal branches of the maxillae approximately level to the 18^th^ maxillary alveoli, and the palatal branches of the maxillae reach posteriorly approximately level to the 20^th^ maxillary alveoli. Posteriorly, the palatines delimit the anterior margins of the internal nares, and posterolaterally form the medial borders of the suborbital fenestrae (Fig. 13). There are two long grooves (one on each side of the central suture) parallel to the anteroposterior axis of the skull (Fig. 8).

The anterior margin of the internal nares is formed by the palatines (that enclose the nares ventrally) and is W-shaped. Posterodorsally the internal nares are enclosed by pterygoid ventral surface (Fig. 13). The surface of the pterygoid is ventrally concave not at the same level as the anterior margin of the choanae (in ventral view it is deeper than palatine surface), and therefore the choanae are posteriorly open.

#### Pterygoids

The pterygoids are exposed on the palatal surface. They are fused into a single element. The palatal surface is large, rounded and deeply concave. Along their anterior margin, the pterygoids contact the palatines. The pterygoids form the lateral and dorsal walls of the choanae (Fig. 13). The internal nares are filled with sediment and it is not possible to determinate the anterior extension of the pterygoids within the nasopharyngeal canal. Along the posterior margin, the pterygoids contact the basisphenoid, and posterolaterally they contact the quadrates (Fig. 13). On both sides of the choana are the pterygoid flanges, which are horizontally orientated and form the posteromedial borders of the suborbital fenestrae. The pterygoid flanges contact the ectopterygoids; however, the sutures between these two elements are unclear (Fig. 13).

#### Parasphenoid

There is a small double pterygoid ridge on the midline (Fig. 13). According to Andrews [10] this structure is the parasphenoid. Whether this determination is correct must await further study and CT-scanning.

#### Ectopterygoids

The ectopterygoids are thin bones, only visible in palatal view, that separate the suborbital fenestrae from the infratemporal fenestrae (Fig. 13). While the sutures are unclear, the ectopterygoids contact the pterygoids medially and the jugals laterally.

#### Suborbital fenestrae

The suborbital fenestrae are elongated and teardrop-shaped, pointing anteriorly. The anteroposterior length of each fenestra is approximately 9 cm and its maximal width 3.5 cm. The fenestrae begin approximately level to the 27^th^ maxillary alveoli, and do not extend anterior to the orbits. Their medial borders are formed by the palatines, while their lateral borders are formed by the maxillae and jugals, and the posterior borders by the ectopterygoids and pterygoids (Figs. 3C, 13).

#### Dentaries

**Figure 14:**
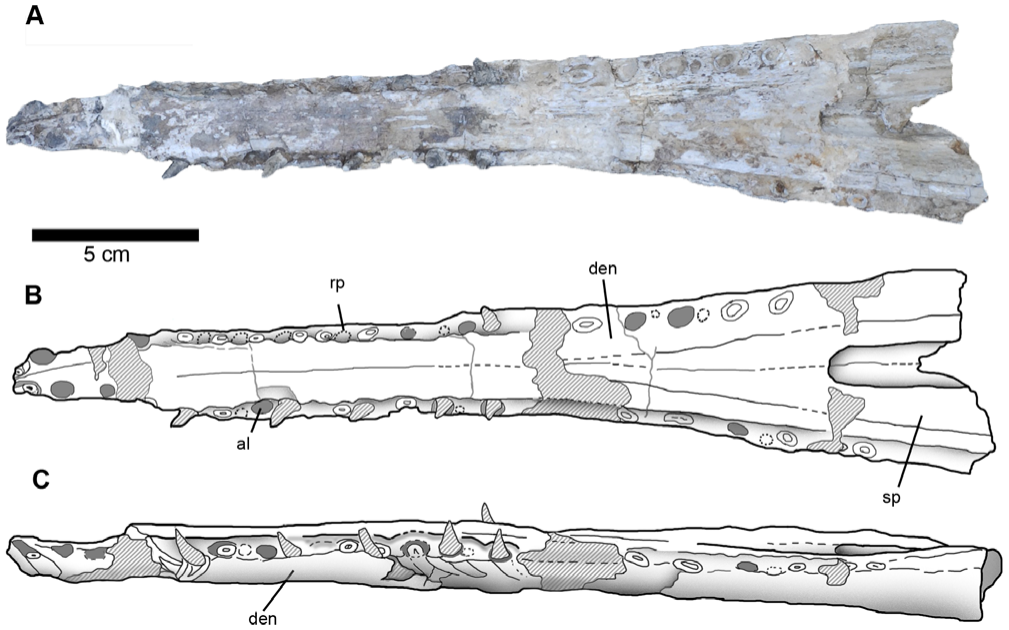
Dentary of *Maledictosuchus riclaensis* gen. et sp. nov. holotype MPZ 2001/130a. Scale bar = 5 cm. A–B, dorsal view photograph and interpretative drawing; C, lateral view. Stripped pattern represents rebuilt mastic surfaces and teeth; grey colored circles represent empty alveoli (teeth are not preserved); circles with broken line represent reception pits. Anatomical abbreviations: al, alveolus; den, dentary; rp, reception pits; sp, splenial.

The dentaries are well preserved, including the mandibular symphysis. The alveolar border and the tip of the symphysis are eroded, and the morphologies of the alveoli are badly preserved. According to Vignaud [28], the number of teeth anterior to the splenial is a good indicator of the total number of teeth. As 21 alveoli are adjacent to the mandibular symphysis, and thirteen anterior to the splenials, we estimate that the dentaries would have had 26–27 teeth. The alveoli are irregularly spaced. The anterior dentary teeth are partially preserved. They are procumbent, conical, without carina and lingually curved. Further back in the tooth row there are incomplete teeth, which preserve regions of enamel. All teeth have apicobasally aligned ridges. The posterior alveoli are larger and labiolingually compressed (Fig. 14).

Furthermore, there are reception pits on the dentary that are in-line with the alveoli (and positioned between them). Most of the reception pits are small and subcircular, but some are pronounced (as grooves), especially between the fifth and sixth, and sixth and seventh right dentary alveoli.

#### Splenials

The splenials form the dorsal-posterior region of the mandibular symphysis, terminating anteriorly level to the 14^th^ dentary alveoli (Fig. 14).

#### Dentition: tooth morphology

The dentition of MPZ 2001/130a is poorly preserved, but the variation in alveolar morphology along the tooth row suggests heterodonty (Fig. 6). The three premaxillary teeth are slightly procumbent, especially PM1. The four anterior-most maxillary alveoli are larger and mediolaterally compressed. At the mid-snout the alveoli are subcircular and subequal in size. The five posterior-most maxillary alveoli are smaller, circular, and very closely packed. Along the dentary tooth row the teeth (visible either on the teeth sections or on the alveoli) become larger and more mediolaterally compressed. The dentary teeth are also procumbent, the anterior teeth are subcircular and outwardly orientated, while the more posterior teeth are mediolaterally compressed and less outwardly orientated. The interalveolar spaces are irregular in size in both the maxilla/premaxilla and the dentary.

All teeth lack carinae (i.e. no keel). The tooth crowns are not enlarged (smaller than 3 cm), lack apicobasal facets on the labial surface (see [1], [81]) and are oval in cross-section. As with all other thalattosuchians (e.g. *Steneosaurus leedsi*: NHMUK PV R3806; *Metriorhynchus superciliosus*: GLAHM V1141; “*Metriorhynchus*” *brachyrhynchus*: NHMUK PV R3804; *Dakosaurus maximus*: SMNS 8203, SMNS 82043), the teeth are single cusped. There is no constriction present at the crown/root junction, but the boundary is evident due to colour and texture.

#### Dentition: type of occlusion

In *Maledictosuchus riclaensis* there are reception pits (caused by the dentary tooth crowns) along the maxillary tooth row, and along the dentary tooth row (caused by maxillary teeth) (Fig. 9). In the upper jaw the reception pits are between the maxillary alveoli. Up to M11 they are offset laterally; and between M18 and M25 (posterior to a region badly preserved) the reception pits are smaller and offset medially (Fig. 6). On the lower jaw, the reception pits are in-line between the dentary alveoli (Fig. 14). This arrangement of reception pits is unique in Metriorhynchidae with the presence of: (1) vertically orientated maxillary teeth (although the anterior-most are slight procumbent), as evidenced by the in-line dentary reception pits; (2) procumbent and outwardly orientated dentary crowns, as evidenced by the laterally-offset reception pits on the anterior-region of the maxillae; and (3) vertically orientated posterior dentary crowns, as evidenced by the medially-offset reception pits on the posterior-region of the maxillae (Fig. 9).

#### Dentition: ornamentation, carinae and wear

**Figure 15:**
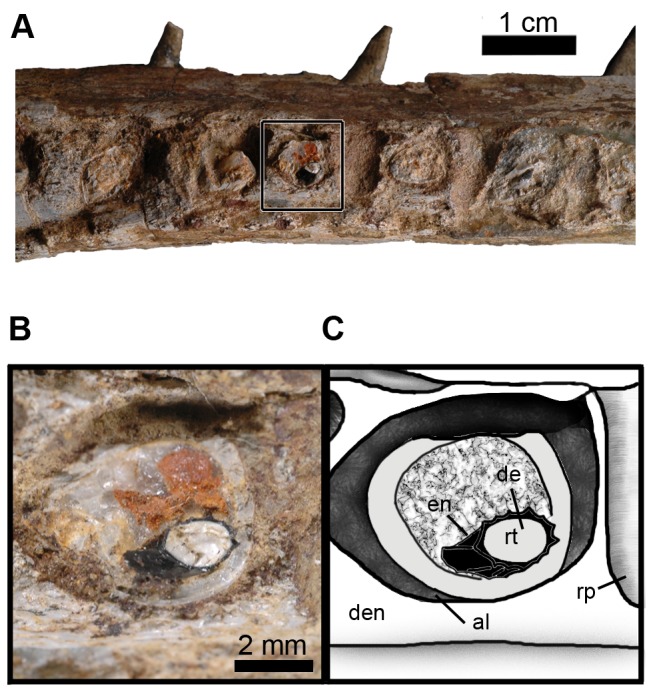
Close-up on a tooth of *Maledictosuchus riclaensis* gen. et sp. nov. holotype MPZ 2001/130a. A, dentary in right lateral view; B–C close-up on a replacement tooth (D8), with well preserved enamel and conspicuous ornamentation. Scale bar A = 1 cm; B–C = 2 mm. Anatomical Abbreviations: al, alveolus; de, dentine; den, dentary; en, enamel; rp, reception pits; rt, replacement tooth.

Tooth enamel ornamentation is composed of apicobasally aligned ridges. The ornamentation on a replacement tooth (D8) is conspicuous and well defined, however on other teeth the ridges are of lower relief. As there are no completely preserved tooth crowns, we cannot determine how the ornamentation varies along the crown or if these ridges are continuous or discontinuous; however, in other metriorhynchid species these ridges do not continue along the entire length of the crown [18], [81]. The enamel ornamentation has no contribution from the underlying dentine (Fig. 15). Split or supernumerary carinae were not found on any tooth, nor is there any evidence for true, or false-ziphodont serrations.

### Phylogenetic Results

**Figure 16:**
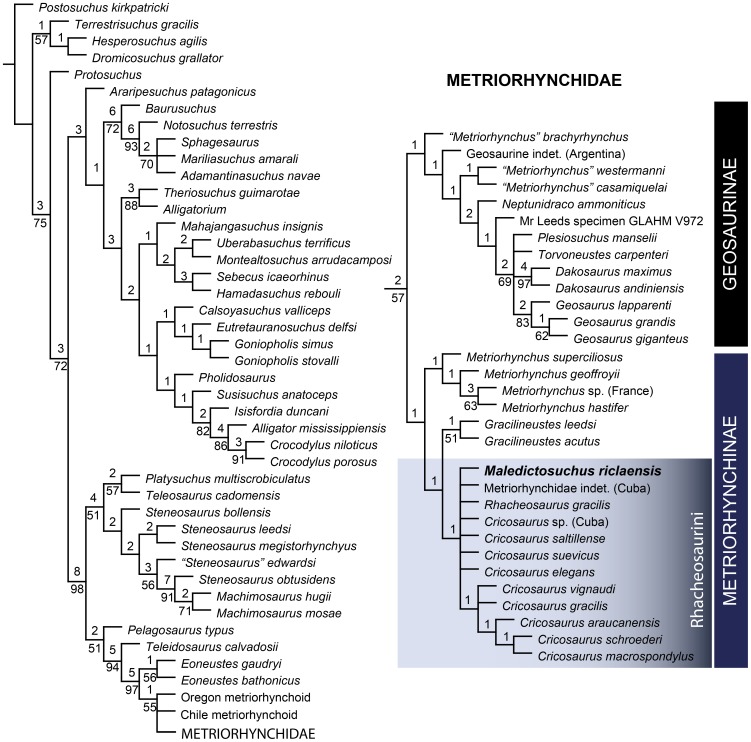
Strict consensus of 8 most parsimonious cladograms of 638 steps, showing the phylogenetic relationships of *Maledictosuchus riclaensis* gen. et sp. nov. within Metriorhynchidae when all characters are unordered. Ensemble consistency index, CI = 0.497; ensemble retention index, RI = 0.857; rescaled consistency index, RC = 0.426. Numbers over lines represent Bremer support values. Numbers under lines represent bootstrap values (only values over 50% represented). The topology is identical to that of Young *et al.* [43]. *Maledictosuchus riclaensis* gen. et sp. nov. is recovered in a large politomy at the base of the tribe Rhacheosaurini.

In the first (unordered) phylogenetic analysis, 8 most parsimonious cladograms were recovered (length = 638, CI = 0.497, RI = 0.857, RC = 0.426). The topology of the strict consensus (Fig. 16) is identical to that of Young *et al.* [43] with the exception of the inclusion of *Maledictosuchus riclaensis*. The unordered analysis results in a large polytomy at the base of Rhacheosaurini just as in Young *et al.* [43]. *Maledictosuchus riclaensis* is recovered within this polytomy.

**Figure 17:**
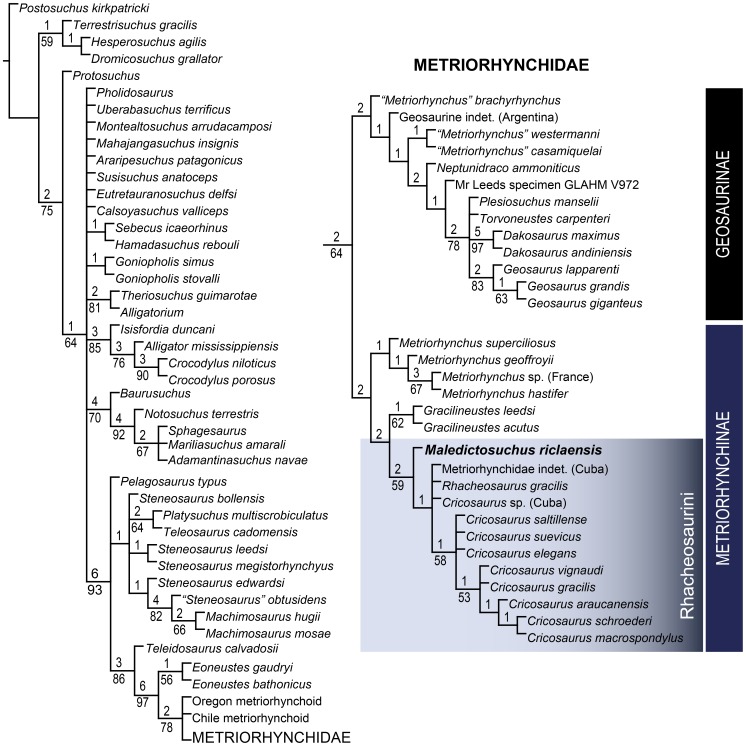
Strict consensus of 194 most parsimonious cladograms of 679 steps, showing the phylogenetic relationships of *Maledictosuchus riclaensis* gen. et sp. nov. within Metriorhynchidae with forty ordered characters (see methods section for complete list of ordered characters). Ensemble consistency index, CI = 0.473; ensemble retention index, RI = 0.860; rescaled consistency index, RC = 0.407. Numbers over lines represent Bremer support values. Numbers under lines represent bootstrap values (only values over 50% represented). Ordering forty multistate characters (see methods) resulted in a loss of resolution among non-metriorhynchids, but significantly improved the resolution within Rhacheosaurini. *Maledictosuchus riclaensis* gen. et sp. nov. is recovered as the basal-most member of this tribe.

The second (ordered) phylogenetic analysis returned 194 most parsimonious cladograms (length = 679, CI = 0.473, RI = 0.860, RC = 0.407). The strict consensus had a significant loss of resolution among the non-metriorhynchid species in the ordered analysis compared to the unordered analysis. However, the phylogenetic relationships within Rhacheosaurini are significantly more resolved, in stark contrast to the polytomy recovered in the unordered analysis. *Maledictosuchus* is found to be the basal-most member of Rhacheosaurini (Fig. 17). The position of *Maledictosuchus* within Rhacheosaurini is well supported as it shares the following rhacheosaurin synapomorphies:

1. Posterior retraction of the external nares (Character 7∶3)
2. Presence of a premaxillary septum (Character 9∶1)
3. Frontal-postorbital suture lower than the intertemporal bar (Character 60∶1)
4. Infratemporal fenestra shorter in length than the orbit (Character 86∶2)
5. Uncarinated teeth (Character 167∶0)
6. Paroccipital processes dorsolaterally inclined (Character 104∶1; see below)

The clade Rhacheosaurini has numerous post-cranial apomorphies. However, *Maledictosuchus riclaensis* only preserves three vertebrae. As such, we cannot determine whether the currently known rhacheosaurin post-cranial apomorphies define the entire clade, or a more inclusive subclade. We have chosen to retain all the post-cranial apomorphies in the diagnosis of Rhacheosaurini (see above), but only future discoveries including the post-cranial skeleton of *Maledictosuchus* or other basal rhacheosaurins can elucidate this subject.

Interestingly, the evolutionary relationships within Rhacheosaurini are now less well-resolved than in previous analyses. All previous incarnations of this dataset that included both *Rhacheosaurus gracilis* and Metriorhynchinae indet. from Cuba (USNM 419640) have found USNM 419640 to be basal to *Rhacheosaurus* [1], [15]. This relationship however, was based on a single character: character 104, in which USNM 419640 is coded [0], whereas all other rhacheosaurins are coded [1] (this character describes the re-orientation of the paroccipital processes from being horizontal [0] to dorsolaterally inclined [1]). *Maledictosuchus*, here found to be basal to both taxa, shares the derived condition with all other Rhacheosaurini (it has the paroccipital process dorsolaterally oriented at a 45 degree angle). This suggests that the horizontal orientation of the paroccipital processes in USNM 419640 is either a reversal or the specimen was incorrectly coded (a possibility as the skull is poorly preserved, especially in that region, and the acid preparation it received further damaged the specimen).

## Discussion

Based on the description and phylogenetic analysis presented above, we show that MPZ 2001/130a is a new taxon. *Maledictosuchus riclaensis* gen. et sp. nov. is characterized by a unique combination of characters (see Diagnosis).

### Comparative anatomy

*Maledictosuchus riclaensis* can be swiftly identified as a metriorhynchid due to its large, ellipsoid orbits that are lateral in position; the enlarged prefrontals that overhang the anterior portion of the orbits; the lachrymals being restricted to the lateral margin of the skull; intertemporal bar that is wider anteriorly (at the frontal contribution) than posteriorly (at the parietal); and the frontal-postorbital suture being “V-shaped” and directed posteriorly. It can be referred to the Metriorhynchinae as it has the following features: long and tubular rostrum; high maxillary tooth count (>20 per maxilla); procumbent teeth; small lachrymals (less than 40% of orbit height); and the palatines have two lateral, non-midline anterior processes.

Within Metriorhynchinae *Maledictosuchus riclaensis* is found to be the basal-most known member of Rhacheosaurini. It shares with all other rhacheosaurins six synapomorphies (see Phylogenetic Results). Interestingly, *Maledictosuchus riclaensis* shares some cranial characteristics with basal metriorhynchines such as *Metriorhynchus* and *Gracilineustes*: greater proportion of the premaxilla posterior to the external nares, and the prefrontals are larger and expand further laterally than other rhacheosaurins. As such, *Maledictosuchus riclaensis* has a mosaic of derived and basal metriorhynchine craniofacial characteristics.

The angle between medial and lateral posterior processes of the frontal is acute (∼60 degrees), this morphology is similar to derived species within *Metriorhynchus* (the Late Jurassic *M. hastifer* [32]), and derived geosaurines (such as *Neptunidraco ammoniticus* and many species within Geosaurini; [17], [43]). However, derived rhacheosaurins, such as *Cricosaurus*, have a more acute angle of ∼40–45 degrees [1], [14], [76]. This again supports *Maledictosuchus riclaensis* as an intermediate between basal metriorhynchines and derived rhacheosaurins. As with derived geosaurines (*Neptunidraco*, *Torvoneustes*, *Geosaurus* and *Dakosaurus*; [17], [43]) the interorbital frontal width of *Maledictosuchus riclaensis* is subequal to the width of one supratemporal fossa. However, in those taxa this morphology is formed by the expansion of the supratemporal fenestrae; by contrast, in *Maledictosuchus riclaensis* the orbits excavate a deeper supraorbital curve than in other metriorhynchines, thus narrowing the frontal.

Compared to the contemporaneous metriorhynchids in the Oxford Clay Formation (OCF) of England *Maledictosuchus riclaensis* is distinct (Table 1). It shares a very high tooth count with *Gracilineustes leedsi* (>30 teeth per maxilla and 18–20 dentary teeth adjacent to the mandibular symphysis). However, *Maledictosuchus riclaensis* lacks the characteristic smooth cranial bones (including a smooth frontal) of *G. leedsi* ([10]; NHMUK Leeds Collection). This high tooth count is noticeably higher than the other OCF species (*Maledictosuchus riclaensis* tooth count has approximately five more teeth than the upper maximum of *Metriorhynchus superciliosus* for both the upper and lower jaws; Table 1). Furthermore, all OCF species have dentition that is bicarinate (continuous carinae on the mesial and distal margins; [18], Table 1), whereas *Maledictosuchus riclaensis* lacks carinae. The geosaurine species can be further distinguished as their carinae possess true denticles [18]. The orientations of the paroccipital processes are horizontal in the OCF species (although as noted above, that could be due to preservation, especially for “*Metriorhynchus*” *brachyrhynchus*), whereas they are dorsolaterally inclined in *Maledictosuchus riclaensis*. The palatine of *Maledictosuchus riclaensis* is also distinct, as it not only possesses the two lateral, non-midline anterior processes seen in *Metriorhynchus superciliosus* and *G. leedsi* [43], but has a third midline process as well (Fig. 8). The geosaurine “*Metriorhynchus*” *brachyrhynchus* only has a midline anterior process of the palatine [10]. The internal nares are divided by a septum, giving the anterior margin of the choanae a “W-shape” with its base directed anteriorly, much like “*Metriorhynchus*” *brachyrhynchus* ([10]; however it must be noted that the palates of OCF metriorhynchids are rarely well-preserved and this morphology could be present in *M. superciliosus* and *G. leedsi*). Finally, the nasal cavity of *Maledictosuchus riclaensis* is divided by a premaxillary septum resulting in the external nares being orientated more laterally than in the OCF species (there are no rhacheosaurins in the OCF).

**Table 1:** List of characters to differentiate between the various Callovian metriorhynchid species (expanded from Young et al., [18] to include *Maledictosuchus riclaensis*).

		Metriorhynchinae			Geosaurinae	
Characters		*Metriorhynchus superciliosus*NHMUK PV R1529, R2032, R2051, R2054, R2055, R6860	*Gracilineustes leedsi*NHMUK PV R3014, R3540	*Maledictosuchus riclaensis*MPZ 2001/130	“*Metriorhynchus*” *brachyrhynchus*NHMUK PV R3804	“Mr Leeds' specimen”GLAHM V972
**Dentition**	Premaxillary alveoli count	3	3	3	3	?
	Maxillary alveoli count	23–27	∼34	30–33	17–18	?
	Dentary alveoli count	20–22	∼34	>21/27?	17	12
	Dentary alveoli adjacent to symphysis	16–17	18–20	21	13	10
	Enamel ornamentation	Conspicuous ornamentation on both surfaces composed of accessory ridges orientated to the apicobasal axis of the crown	Ornamentation largely inconspicuous to the naked eye. Composed of apicobasal ridges that are low and become rarer near the apex	Conspicuous ornamentation on both surfaces composed of accessory ridges orientated to the apicobasal axis of the crown	Labial surface lacks conspicuous ornamentation, the lingual surface has possesses short but high apicobasal ridges	Ornamentation inconspicuous, apicobasal ridges on both surfaces are restricted to the base of the crown, and are low, well-spaced and very short
	Carinae	Present – bicarinate	Present – bicarinate	Absent	Present – bicarinate	Present – bicarinate
	Microscopic denticles	No	No	No	Yes	Yes
**Cranium**	Frontal surface texture	Ornamented, shallow to deep elliptical pits. These can fuse to become deep, radial grooves in some specimens	Smooth	Ornamented, shallow grooves arranged in a radial pattern and elliptical pits	Ornamented, shallow to deep elliptical pits. These can fuse to become deep, radial grooves in some specimens	?
	Prefrontal surface texture	Ornamented, large shallow to deep elliptical pits	Smooth	Ornamented, small shallow elliptical pits	Ornamented, large shallow to deep elliptical pits	
	Squamosal overlap of the paroccipital process (in dorsal aspect)	Small, the paraocciptal process projects further posteriorly than the squamosal	Small, the paraocciptal process projects further posteriorly than the squamosal	Small, the paraocciptal process projects further posteriorly than the squamosal	Extensive, projecting further posteriorly than the paraocciptal process itself	?
	Paroccipital process orientation	Horizontal	Horizontal	Dorsolaterally inclined	?Horizontal (possibly taphonomic)	?
	Shape of the prefrontal expansion over the orbits (in dorsal aspect)	Lateral margin forms a convex curve	Lateral margin forms a convex curve	Lateral margin forms a convex curve	Lateral margin forms a 90 degree angle giving it a distinct triangular shape	?
	Palatine rostral development along the midline	Adjacent to 18^th^ maxillary alveoli	Adjacent to 19^th^ maxillary alveoli	Adjacent to ∼18^th^ maxillary alveoli	Adjacent to 10^th^ maxillary alveoli	?
	Palatine with two non-midline anterior processes (one on either side of the mid-line)	Yes, both separate from the mid-line and the maxilla alveolar border. Terminate adjacent to the 15^th^ maxillary alveoli	Yes, both separate from the mid-line but lateral margin sutures with the maxilla alveolar border. Terminate adjacent to the 8^th^ maxillary alveoli	One midline and two non-midline anterior processes, both separate from the maxilla alveolar border. Begin (posteriorly) ∼20^th^ and terminate adjacent to the ∼18^th^ maxillary alveoli	No	?
**Mandible**	Mandibular symphysis depth relative to mandible length	∼4.5–6%	∼4%	?	∼7–8%	∼5%
	Dentary tooth row ventrally displaced relative to jaw joint	No	No	?	No	Yes
	Coronoid process ventrally displaced relative to jaw joint	No	No	?	No	Yes
	Presence of reception pits along either tooth row	No	No	Yes, between the maxillary alveoli and dentary alveoli	Yes, on the lateral margin of the dentary	Yes, sagittal plane between the dentary alveoli (D7–D12)
**Vertebrae**	Atlas hypocentrum length	Hypocentrum length subequal to odontoid process length	Hypocentrum length subequal to odontoid process length	?	Hypocentrum length greater than odontoid process length	?
	Cervical centra dimensions	Centrum length shorter than their height	Centrum length shorter than their height	?	Centrum length greater than their height	Centrum length and height subequal
**Pectoral girdle**	Deltopectoral crest shape	Distinctly triangular	Gentle convex curve	?	Distinctly triangular	Distinctly triangular
	Relative size of humerus and scapula	Scapula shorter than humerus	Humerus shorter than scapula	?	Scapula shorter than humerus	?
**Pelvic girdle**	Dorsal border of ilium acetabulum contribution	Orientated ventrally	?	?	Orientated ventrally	Orientated horizontal
	Ilium dorsal border relative to ilium acetabulum contribution	Terminates approximately level to mid-point of acetabulum	?	?	Terminates approximately level to mid-point of acetabulum	Terminates prior to acetabulum
	Ischium acetabulum contribution	Laterally-directed, slightly concave semi-circle	?	?	Laterally-directed, slightly concave semi-circle	Laterally-directed, deeply concave semi-ellipsoid
	Ischium acetabulum contribution and anterior process for the pubis	Clearly separated	?	?	Clearly separated	Connected by a medially-directed convex ridge
	Ischium process for the pubis	Articulation facet is rounded and smooth	?	?	Articulation facet is rounded and smooth	Articulation facet is complex (hills and troughs)

Data from the Leeds Collections of NHMUK (NHMUK, Natural History Museum, London, United Kingdom) and GLAHM (Hunterian Museum, Glasgow, United Kingdom). Note, we do not currently consider NHMUK PV R1994 and NHMUK PV R2039 to belong to “*Metriorhynchus*” *brachyrhynchus*. These specimens and the synonymy between “*M.*” *brachyrhynchus* and *Suchodus durobrivensis* is currently under review by one of us (MTY).

### Marine specialisation in Rhacheosaurini

Within Rhacheosaurini there is a suite of cranial characteristics that Young *et al.* [14] hypothesized were linked to increasing marine specialization: 1) external nares that are divided by a midline premaxillary septum, 2) posterodorsal retraction of the external nares, and 3) posterior reorientation of the lateral processes of the frontal, and the consequent formation of an acute angle between the medial and lateral processes of the frontal. The divided external nares is hypothesized to be an adaptation for more efficient respiratory airflow [20], while the posterodorsal retraction of the external nares is observed in clades with sustained swimmers (e.g. ichthyosaurs and cetaceans; see [82] and references therein). The re-orientation of the lateral processes of the frontal is involved in cranial streamlining [14], [76]. These three characteristics are, to a greater or lesser degree, exhibited by *Maledictosuchus riclaensis*, and are absent in contemporaneous OCF species (Table 1; [10]; [14]; NHMUK Leeds Collection). Prior to the discovery of *Maledictosuchus riclaensis*, the oldest known metriorhynchid that exhibited this suite of adaptations was the Late Kimmeridgian *Cricosaurus suevicus* [11], [70]. As such, the evolution of greater marine specialization and the shift towards a mesopelagic, open-sea, lifestyle began approximately 10 Ma earlier than previously supposed during the Middle Jurassic (Middle Callovian).

### Specialised piscivory/teuthophagy in Rhacheosaurini

Recent analyses based on the evolution of skull strength in metriorhynchids have shown that rhacheosaurins had low strength skulls not suited to feeding on large-bodied prey [14], [16], [83]. Furthermore, most species within this group have an elongate, tubular rostrum, with procumbent and non-carinated dentition, and a high overall tooth count. However, Tithonian specimens of *Cricosaurus saltillense* [21] and *Cricosaurus* sp. [19], and the Valanginian species *Cricosaurus macrospondylus* [20], had carinated and more vertically orientated dentition. In most rhacheosaurins, the lack of cutting edges on the dentition, the teeth orientated forward in the mouth and a tubular low-strength rostrum is in marked contrast to the contemporaneous subclade Geosaurini which had: high-strength brevirostrine-mesorostrine rostra, carinated and serrated dentition, vertically orientated tooth crowns positioned to maximize shear, a low overall tooth count (<15 teeth per maxilla and dentary) and an increased gape [14], [16], [18], [43], [81], [83], [84]. The stark difference in craniodental morphologies between these two subclades has led to hypotheses of niche partitioning and ecological specialization, with rhacheosaurins more suited to feeding on small, fast moving prey [14], [16], [83], [85]. While basal metriorhynchines such as *Metriorhynchus superciliosus* and *Gracilineustes leedsi* are similar in having a high tooth count and a tubular rostrum (Table 1), *Maledictosuchus riclaensis* is the oldest known metriorhynchine with non-carinated teeth.

Moreover, as with other rhacheosaurins, *Maledictosuchus riclaensis* shares a more verticalised squamosal, and dorsolaterally inclined paroccipital processes [14]. We hypothesise that these adaptations would have resulted in increased fibre lengths for the *M. depressor mandibulae*, and thus increased jaw-closing velocity. Such an adaptation would have facilitated capture of small, fast-moving prey.

The occlusion mechanism of *Maledictosuchus riclaensis* is unique among metriorhynchids, as it combines tightly-packed vertically orientated/slightly procumbent upper jaw teeth with procumbent/outwardly orientated lower jaw teeth. Coupled with the tubular rostrum and non-carinated teeth, these features suggest that this species had an occlusion mechanism adapted for impaling and securely holding small prey items (such as fish and cephalopods). Another Callovian species (see Table 1), the “Mr Leeds' specimen” (GLAHM V972, the new generic name is currently in press), also has maxillary teeth that are ‘in-line locking’ (with in-line reception pits between the dentary alveoli); however, this taxon has enlarged dentition that is both carinated and serrated, and has an increased gape [18]. A third Callovian species, “*Metriorhynchus*” *brachyrhynchus*, has pronounced reception pits on the lateral margins of the dentary created by the upper jaw dentition [18]. It would appear that the high species diversity of metriorhynchids in the Callovian of Europe co-occurs with a high diversity of craniodental morphologies and distinct occlusion mechanisms, thereby enabling niche partitioning and ecological/trophic specialisation.

### Callovian diversification of metriorhynchids

Based upon recent re-examination of fossil specimens from the Callovian of Europe, the key craniodental adaptations that characterize the derived Geosaurini and Rhacheosaurini evolved during the Middle Jurassic. The description of the “Mr Leeds' specimen” (GLAHM V972) has highlighted that the adaptations that underpinned the success of the large-bodied macrophagous Geosaurini in the Late Jurassic and Early Cretaceous first appeared during the Callovian [18]. Here, we have shown that the craniodental morphologies that underpinned the success of Rhacheosaurini in the Late Jurassic and Early Cretaceous (from increasing marine specialization to adaptations for feeding on small-bodied prey) also first appeared during the Callovian.

Of all the marine Callovian formations of Europe, the OCF is the best represented and most studied. The ecosystem is dominated by *Metriorhynchus superciliosus* (with 50+ specimens), while *Gracilineustes leedsi* is known from approximately 12 specimens ([10]; NHMUK Leeds Collection and GLAHM Leeds Collection). The short/broad-snouted species “*Metriorhynchus*” *brachyrhynchus*, “Mr Leeds' specimen” and *Suchodus durobrivensis* are collectively known from approximately 20 individuals [18]. Note that the validity of these species is currently being investigated by one of us (MTY). In the OCF *Maledictosuchus riclaensis* is absent. In the Oxford Clay Sea, basal rhacheosaurins and geosaurins were curiously rare/absent. It would appear that the feeding/marine adaptations both groups evolved did not confer immediate selective advantage upon them, and it took until the Late Jurassic for these two subclades to dominate in Western Europe [14]. However, another possibility is that the sister taxa and basal members of the subclades Geosaurini and Rhacheosaurini were more common, and radiated, in deeper water. Possible evidence for this is *Neptunidraco ammoniticus*, a Bajocian/Bathonian taxon known from the Tethys Ocean. *Neptunidraco* is the oldest known metriorhynchid with a streamlined skull (posterolaterally re-orientated lateral processes of the frontal); with a skull more streamlined than most younger Callovian species [17].

Whether the Late Jurassic–Early Cretaceous dominance of Rhacheosaurini and Geosaurini was due to selective advantage and out-competing more basal metriorhynchids or due to opportunistic replacement after climatic shifts (e.g. of sea temperature, sea level and/or marine fauna) currently cannot be determined.

## Conclusions

Upon re-examination of an understudied skull from the Callovian of Spain, the morphological and taxonomic diversity of Middle Jurassic metriorhynchids is found to be greater than previously realised. The phylogenetic analysis presented here clearly shows that the new species we describe is the basal-most member of Rhacheosaurini, a lineage of increasingly mesopelagic piscivores. Herein we emend the diagnosis for this clade.

*Maledictosuchus riclaensis* gen. et sp. nov. represents a morphological intermediate between basal metriorhynchines and the derived Late Jurassic members of Rhacheosaurini, possessing a mosaic of basal and derived craniodental characteristics. While it is a morphologically intermediate, it was contemporaneous in age with the basal metriorhynchines of Europe (*M. superciliosus* and *G. leedsi*). *Maledictosuchus riclaensis* is the oldest known rhacheosaurin, and is currently the oldest and best preserved metriorhynchid specimen from the Iberian Peninsula. Alongside with the recent description of the “Mr Leeds' specimen” from the Callovian of England, we now have evidence that the evolution of derived metriorhynchids (subclades Geosaurini and Rhacheosaurini) began during the Middle Jurassic. While their craniodental morphologies evolved in a mosaic manner, the key adaptations that enabled their evolutionary radiations in the Late Jurassic (e.g. cranial streamlining, feeding related dentition characters) first appear approximately 10 million years earlier.

## Supporting Information

Text S1 **Character by taxon matrix used in the phylogenetic analysis (tnt file).**
(TXT)
